# Concentration of circulating miRNA-containing particles in serum enhances miRNA detection and reflects CRC tissue-related deregulations

**DOI:** 10.18632/oncotarget.12205

**Published:** 2016-09-23

**Authors:** Abdou ElSharawy, Christian Röder, Thomas Becker, Jens K. Habermann, Stefan Schreiber, Philip Rosenstiel, Holger Kalthoff

**Affiliations:** ^1^ Institute of Clinical Molecular Biology, Christian-Albrechts-University, Kiel, Germany; ^2^ Institute for Experimental Cancer Research, Christian-Albrechts-University, Kiel, Germany; ^3^ Department of General Surgery, Visceral, Thoracic, Transplantation and Pediatric Surgery, University Hospital Schleswig-Holstein, Campus Kiel, Kiel, Germany; ^4^ Section for Translational Surgical Oncology and Biobanking, Department of Surgery, University of Lübeck and University Hospital Schleswig-Holstein, Campus Lübeck, Lübeck, Germany; ^5^ Clinic for Internal Medicine I, University Hospital Schleswig-Holstein, Campus Kiel, Kiel, Germany; ^6^ Faculty of Sciences, Division of Biochemistry, Department of Chemistry, Damietta University, New Damietta City, Egypt

**Keywords:** serum biomarker, extracellular microvesicles, miRNA-containing particles, colorectal neoplasms and inflammatory bowel disease, strand-specific miRNA deregulation

## Abstract

The emerging potential of miRNAs as biomarkers for cancer detection demands parallel evaluation of strategies for reliable identification of disease-related signatures from easily accessible and pertinent body compartments. Here, we addressed whether efficient concentration of circulating miRNA-carrying particles is a rationale for miRNA biomarker discovery. We systematically compared miRNA signatures in 93 RNA preparations from three serum entities (whole serum, particle-concentrated, and particle-depleted fractions) and corresponding tissue samples from patients with colorectal cancer (CRC) as a model disease. Significant differences between whole sera and particle-concentrated serum fractions of CRC patients emerged for 45 of 742 tested miRNAs. Twenty-eight of these 45 miRNAs were differentially expressed between particle-concentrated serum fractions of metastatic CRC- and healthy individuals. Over half of these candidates (15 of 28) showed deregulations only in concentrated serum fractions, but not in whole sera, compared to the respective controls.

Our results also provided evidence of a consistent downregulation of miR-486 and miR-92a, and further showed a possible “strand-specific” deregulation of extracellular miRNAs in CRC. More importantly, most of the identified miRNAs in the enriched sera reflected the patterns of the corresponding tumor tissues and showed links to cancer-related inflammation. Further investigation of seven serum pools revealed a subset of potential extracellular miRNA candidates to be implicated in both neoplastic and inflammatory bowel disease.

Our findings demonstrate that enrichment and sensitive detection of miRNA carriers is a promising approach to detect CRC-related pathological changes in liquid biopsies, and has potential for clinical diagnostics.

## INTRODUCTION

It is becoming increasingly clear that circulating miRNAs represent suitable biomarkers for a broad spectrum of diseases [1, 2], e.g. for cancer. In addition, they are important mediators of cell-cell communications [3]. This class of small noncoding RNAs controls gene expression by different mechanisms and, when released by cancer cells, may induce a pro-metastatic inflammatory response [4]. A major challenge is the establishment of a sensitive, specific and robust detection strategy of disease-related miRNA signatures in patient's blood samples.

Several recent studies characterized circulating miRNAs as protein-bound [5, 6] and utilized suitable immunoprecipitation protocols for isolation of such ribonucleo-proteins [7]. Other studies demonstrated that miRNAs are contained and transported in extracellular microvesicles (EVs) or exosomes present in biological fluids [8, 9]. The key arguments for considering EV miRNAs in biomarker discovery are that (i) these EVs have complex biochemical structures that protect their contents and resemble the cells they originated from; (ii) profiling EVs cargo (RNA, proteins, etc.) can be a window to the genetic status of individual tissues, providing important biological/functional insights, and can be regarded as a fingerprint of a disease; and (iii) understanding these mechanisms in this new area of research is of great diagnostic, prognostic and therapeutic potential for human diseases [10–12].

Many reports also investigated whole blood as an easily accessible compartment for detection of extracellular miRNAs [1, 13, 14]. Yet, other studies have argued against this because of the interferences of blood cell-miRNAs and possible hemolysis as an additional source of unwanted miRNA [15]. Moreover, the actual origin and function of extracellular miRNAs, as either “non-specific by-products” or “on-purpose messengers”, remain under debate [16].

These studies demonstrate the challenges of investigating miRNA (signatures) and might explain why other studies analyzed miRNAs in the tissue of interest directly, rather than extracellular miRNA [17]. Yet, non-invasive early detection, through standardized (pre)analytical procedure with a focus on EV-miRNAs, is currently needed [18].

In the light of this information, together with the heterogeneity of the reported studies (analysis of whole blood, serum/plasma, fractions thereof, or tissue samples; detection by microarray, qPCR, or sequencing; data normalization; etc), it is not surprising that little overlap has emerged between the results of different biomarker studies for the same disease.

These observations reflect the need to evaluate suitable strategies for reliable identification of miRNA disease-related signatures from easily accessible biofluids. We therefore followed a comprehensive approach, which integrates an efficient method for isolation of the “bulk” of miRNA carriers prior to sensitive qPCR profiling, to compare miRNA expression patterns in three serum entities (whole serum, particle-concentrated, and particle-depleted fractions), and tissue samples from the same patients with colorectal cancer (CRC) as a model disease.

## RESULTS

### Discovery of miRNA blood biomarker candidates for CRC in whole sera compared to particle-concentrated serum fractions

**Figure 1:**
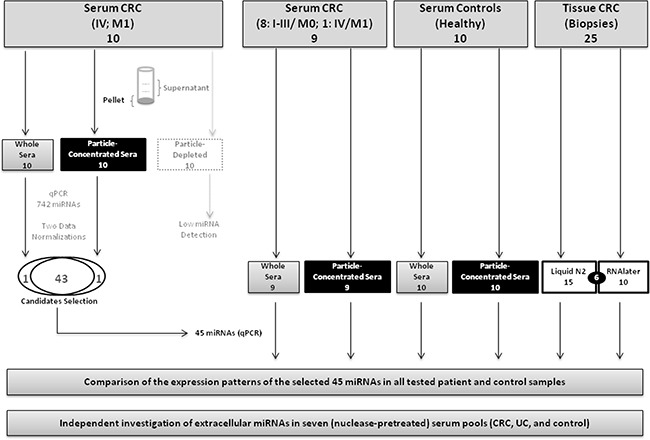
Study design and experimental setup This figure shows the types of the 93 RNA samples tested in this study. Using a 742-miRNA qPCR panel, we first identified 45 differentially expressed miRNAs, which we further investigated in 63 RNA samples from an independent CRC patients' and healthy controls' sera as well as paired tumor tissue samples. We additionally analyzed extracellular miRNA patterns, using another EVs isolation method, in seven (nuclease-pretreated) serum pools of colorectal cancer (CRC), ulcerative colitis (UC) and healthy individuals.

The strategy of our study to identify CRC-related miRNA biomarkers in the blood is depicted in Figure 1. A general focus was laid on the possible impact of a robust and easy-to-perform pre-analytic concentration of miRNA-carrying particles in favor of an improved detection of CRC-specific miRNA signatures in the blood. Comparison of the detectability of 742 miRNAs in 30 RNA preparations from different serum fractions (Figure 1) of 10 CRC patients (UICC stage IV, compare Supplementary Table S2a) was performed on Exiqon *miRCURY LNA^TM^ Universal RT miRNA PCR Human panels-I-and-II* and revealed substantial differences depending on the pre-analytic processing of the sera. The total number of detected miRNAs was higher in the particle-concentrated fractions, than in the whole sera and corresponding particle-depleted fractions (~150-250, ~80-120, and ~20-50 miRNAs, respectively) (Supplementary Figure S1).

Following PCR-panel processing, normalization and filtering, >40 miRNAs were detected on average in all whole sera and particle-concentrated fractions, but only 11 miRNAs in the particle-depleted fractions. Statistical analyses showed a separation and clustering of the matched particle-concentrated and supernatant fractions (Supplementary Figures S2 and S3). Each of two normalization approaches employed (Supplementary Data), yielded 44 statistically significant miRNA candidates, which showed differential expression between whole sera and the corresponding particle-concentrated fractions (Supplementary Table S1). Of all miRNAs analyzed, 43 candidates with consistent detectability after both normalizations, plus two unique miRNAs (miR-143, miR-423), were considered for the subsequent analysis.

In addition, we assessed the effect of different RNA preservation methods for tissue miRNA detection (details in Supplementary Data, Supplementary Tables S5 and S6, and Supplementary Figure S4). Accordingly, we considered only the data of the tissues preserved in liquid nitrogen (n=15) in our downstream analyses in order to avoid additional confounding factors.

### Improved detection of miRNA biomarker candidates after enrichment of miRNA carriers from serum

Further analysis and comparison of expression of the selected 45 miRNA candidates in 63 independent RNA preparations, from serum and corresponding tumor tissue samples (Supplementary Table S2b; Figure 1), resulted in 22 differentially expressed miRNAs in patients' particle-concentrated sera (Table 1; Supplementary Table S3), but only 13 miRNAs in patients' whole sera (Supplementary Table S4).

**Table 1:** Particle-Concentrated serum miRNA patterns reflect CRC tissue-related expression deregulations

		I. Particle-concentrated sera (CRC *vs.* controls)		II. Particle-concentrated sera *vs.* tissue (all CRC)		Consistent expression of I & II (15/22; 68%)	III. Tissue (CRC) *vs.* particle-concentrated sera (controls)		Consistent expression of I & III (20/22; 91%)
Expression pattern with respect to CRC	miRNA candidates	Fold change^a^	Adjusted P-value	Fold change^b^	Adjusted P-value		Fold change^c^	Adjusted P-value	
**↑↑** **Particle-concentrated Sera & tissue** (n=10)	miR-22-3p	2.673	3.04×10^−05^	0.311	*0.4852*	**Yes**	2.362	2.02×10^−06^	**Yes**
	miR-21-5p	4.766	5.65×10^−05^	−1.123	*0.2398*	**Yes**	5.889	7.05×10^−07^	**Yes**
	miR-29c-3p	2.482	5.65×10^−05^	−1.738	0.0059	**Yes**	4.221	7.53×10^−08^	**Yes**
	miR-101-3p	3.334	8.91×10^−05^	1.345	*0.1126*	**Yes**	1.989	0.0207	**Yes**
	miR-23a-3p	0.673	0.0002	−0.882	0.0005	**Yes**	1.555	2.62×10^−07^	**Yes**
	miR-23b-3p	1.259	0.0004	−2.766	1.16×10^−08^	**Yes**	4.026	4.60×10^−12^	**Yes**
	miR-423-5p	2.516	0.0010	1.053	*0.1126*	**Yes**	1.463	0.0018	**Yes**
	miR-24-3p	1.473	0.00293	−1.684	0.0019	**Yes**	3.157	1.66×10^−09^	**Yes**
	let-7f-5p	1.328	0.0095	−1.065	*0.1326*	**Yes**	2.394	0.0022	**Yes**
	miR-125b-5p	1.243	0.0454	−5.460	5.17×10^−08^	**Yes**	6.704	8.05×10^−13^	**Yes**
**↑ Particle-concentrated sera only** (n=3)	miR-22-5p	6.130	7.40×10^−05^	5.360	0.0001	No	0.770	0.1898	**Yes**
	miR-223-3p	2.151	0.0051	2.292	0.0083	No	−0.140	0.8114	**Yes**
	miR-320b	0.795	0.0134	1.103	0.0030	No	−0.307	0.3353	**Yes**
**↑Particle-concentrated sera & ↓tissue** (n=2)	miR-335-5p	1.832	0.0002	3.241	2.48×10^−09^	No	−1.408	0.0024	No
	miR-144-3p	3.330	0.0004	6.713	6.45×10^−07^	No	−3.3820	0.0019	No
**↓↓ Particle-concentrated sera & tissue** (n=5)	miR-486-5p	−2.119	0.0001	7.347	1.14×10^−14^	**Yes**	−9.466	3.35×10^−16^	**Yes**
	miR-93-5p	−0.951	0.0003	0.489	0.0637	**Yes**	−1.441	5.70×10^−07^	**Yes**
	miR-92a-3p	−1.1269	0.0026	−0.023	*0.9485*	**Yes**	−1.103	0.0085	**Yes**
	miR-146a-5p	−0.777	0.0030	0.492	*0.1820*	**Yes**	−1.270	0.0009	**Yes**
	miR-221-3p	−1.325	0.0064	−0.057	*0.9485*	**Yes**	−1.268	0.0036	**Yes**
**↓ Particle-concentrated sera only** (n=2)	let-7d-3p	−2.006	0.00070	−1.549	0.0049	No	−0.456	0.1984	**Yes**
	miR-342-3p	−1.205	0.0064	−1.527	0.0003	No	0.321	0.3828	**Yes**

↑: overexpression; ↓: lower expression;

a Fold changes (ddC_q_ values of particle-concentrated CRC sera minus those of the controls); positive values: upregulation in particle-concentrated CRC sera;

b Fold changes (ddC_q_ values of particle-concentrated CRC sera minus those of the CRC tissue); positive values: upregulation in the particle-concentrated CRC sera or downregulations in the CRC tissue;

c Fold changes (ddC_q_ values of CRC tissue minus those of the particle-concentrated sera of controls); positive values: upregulation in the CRC tissue. More details can be found in Supplementary Data (pp. 3-4).

**Figure 2:**
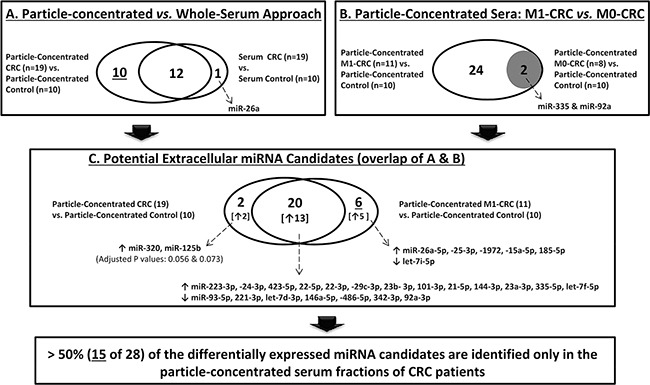
Differentially expressed extracellular miRNAs in CRC This figure illustrates the different comparisons performed to identify differentially expressed miRNAs in CRC. Remarkably, more than half, 15 of 28 miRNA candidates (10 from A and 5 from C, after excluding miR-26a that was identified in the whole sera), were exclusively identified in CRC particle-concentrated serum compartment. (↑: higher expression; ↓: lower expression).

We observed an overlap of 12 detected miRNAs between whole sera and the particle-concentrated fraction. However, 10 miRNAs were only identified in the particle-concentrated fractions (Figure 2). Interestingly, one miRNA (miR-26a) showed elevated expression only in the whole sera. Yet, miR-26a was also emerged in the particle-concentrated fractions when comparing enriched serum fractions of metastatic CRC patients to those of controls (Figure 2c).

### miRNA expression in particle-concentrated sera mirrors deregulation of tumor tissues

**Figure 3:**
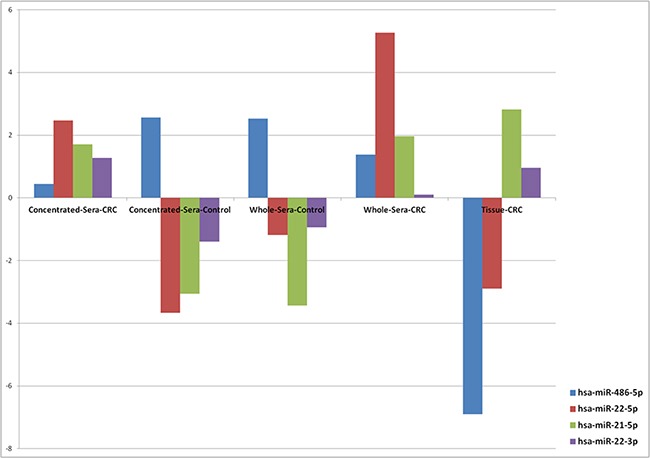
Expression trends of miRNAs in serum and CRC tissue samples This figure shows examples of the results of ANOVA analysis of four miRNA candidates across five different sample types. Common low expression of miR-486-5p (P-value, 3.39×10^−32^) and upregulation of miR-21-5p (P-value, 1.64×10^−10^) were observed in CRC. A “strand-specific” expression was observed for miR-22.

To evaluate whether miRNA patterns in the particle-concentrated fractions reflect deregulations of miRNA expression in the corresponding tumors, we examined the detectability of the 45 miRNA candidates (I) in the particle-concentrated sera of patients versus controls, (II) in the particle-concentrated sera of CRC patients versus their corresponding tumor tissues, and (III), in these CRC tissues versus particle-concentrated sera of control individuals. The twenty two miRNA candidates with differential expression in patients' particle-concentrated sera are listed in Table 1. The detection of more than two-third (15/22) of these miRNAs was consistent in CRC-patients' particle-concentrated sera and the matched tumor tissue samples (indicated by “Yes” in Table 1/columns 7 and 10). Ten of these 15 miRNAs were simultaneously overexpressed, whereas five showed consistently decreased expression (marked grey in Table 1). Five of the 10 overexpressed miRNAs (miR-29c-3p, -23a-3p, -23b-3p, -24-3p, and -125b-5p) showed an elevated expression in tissues than in the matched particle-concentrated sera. Two of these five miRNAs (miR-23a-3p, miR-125b-5p) were exclusively identified in the particle-concentrated compartment (Table 1). Similarly, four of the five miRNAs with reduced expression, in both CRC-patients' particle-concentrated sera and the matched tumor tissue samples (i.e., all except for miR-486-5p), were exclusively identified in CRC-patients' particle-concentrated sera, but not in the whole serum samples. However, the remaining miRNAs (7/22; Table 1) showed inconsistent expression between the particle-concentrated fractions and corresponding tissues (Supplementary Data). The ANOVA analysis underlined potential candidates with coordinated expressional changes of particle-concentrated sera and tissue miRNAs (Figure 3; Supplementary Table S7).

### miRNA *in silico* analyses and relation to cancer and inflammatory pathways

*In silico* analyses of the identified 22 miRNAs (Table 1), using the miEAA tool, showed significant overrepresentation in the context of exosomes (17 miRNAs), and many diseases, such as carcinoma/adenocarcinoma (22 and 17 miRNAs), neoplasm/metastasis (21 miRNAs), inflammation (16 miRNAs), and colonic-neoplasms (10 miRNAs). Using other analysis options of miEAA, designated “Organs-miRWalk” and “pathway-miRWalk”, numerous significant overrepresentations of the identified 22 miRNAs in different organs/tissues and inflammation-/cancer-related pathways were found (Supplementary Table S8; Supplementary Data). A review of the current literature further confirmed the potential interaction of at least 16 of these 22 miRNAs (73%; Supplementary Table S9) with key inflammatory-/cancer-related factors, e.g., IL6, STAT3 and NF-κB.

### Extracellular miRNA candidates of pathophysiological relevance in metastatic CRC

To address whether miRNA signatures in particle-concentrated serum fractions associate with CRC progression, we compared the expression pattern of the tested 45 miRNAs in patients with distant metastasis (M1) and individuals without metastasis (M0) to those of the controls. While 26 differentially expressed miRNAs were identified in the first comparison (CRC-M1 *vs*. controls), only 2 miRNAs (miR-335, miR-92a) were emerged in the second comparison (CRC-M0 *vs*. controls). These two miRNAs were also detected in the first 26-miRNA panel (Figure 2). Notably, 20 of the 22 miRNAs identified in the particle-concentrated sera were from these 26 miRNAs. Here, five of the six non-overlapping miRNAs between the two comparisons (Figure 2) would have been missed if we would have only analyzed whole sera. Altogether, these results underline that an additional 15 miRNAs (>53%) were identified from the patients' particle-concentrated sera. Moreover, the expression data of these 26 miRNAs revealed similar coordinated patterns of expression in CRC-M1 particle-concentrated sera and paired tissue samples, with a previously reported link to inflammatory-/cancer-related factors (Supplementary Tables S10 and S11).

### Consistent expression patterns of 14 extracellular miRNAs in both CRC and IBD

**Figure 4:**
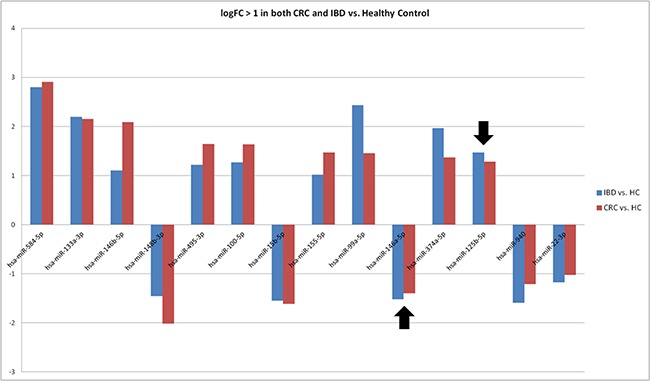
Consistent differential expression of 14 miRNAs in EV-Enriched sera fractions of both CRC and IBD This figure demonstrates consistent overexpression of nine miRNAs and low expression of five miRNAs revealed in nuclease pre-treated, EV-enriched serum pools of CRC and IBD compared to a healthy control pool. The black arrows point to the elevated expression of miR-125b-5p and reduced expression of miR-146a.

In order to explore which extracellular miRNAs are interrelated in their expression from the clinical samples of patients with CRC and inflammatory bowel disease (IBD), we also investigated miRNA patterns of EVs isolated from pooled sera of both CRC and ulcerative colitis (UC-)patients (six patients per pool). Seven serum pools (3 pools each for CRC and UC and one pool from healthy individuals) were pretreated with RNase and DNase and screened utilizing an Exiqon *miRCURY LNA^TM^ Universal RT miRNA PCR Human panel I* comprising 372 miRNA assays. Out of all 77 miRNAs detectable in all disease pools, only miR-30b was significantly differentially expressed (P=0.012) between CRC and UC pools. miR-30b was overexpressed in CRC compared to UC and healthy control pools. Interestingly, 14 miRNAs out of these 77 miRNAs showed differential expression of more than 2-fold in the comparisons of both CRC *vs*. healthy and UC *vs*. healthy pools. As shown in Figure 4, these 14 miRNAs were consistent in their expression between the two disease states compared to the healthy control pool. Notably, two of these miRNAs, miR-125b-5p (with higher expression) and miR-146a-5p (with lower expression) showed similar expression tendencies, as previously shown in our matched CRC serum and tissue samples (Table 1).

## DISCUSSION

In the present study, we demonstrated for the first time that the concentration of circulating (EVs and non-EVs) miRNA carriers based on steric exclusion, when followed by qPCR profiling, allowed a more reliable identification of CRC-related miRNAs. This approach further enabled the disclosure of candidates with potential functional relevance and allowed a better reflection of disease tissue-related patterns.

### Towards a comprehensive analysis of extracellular miRNAs

The use of miRNAs as biomarkers in liquid biopsies is rapidly evolving. Yet, intra-and inter-experimental consistency and routine laboratory standards remain challenging [19]. Additionally, blood poses heterogeneous and poorly defined miRNA carriers and EVs composition [20]. Moreover, the biological source of extracellular miRNAs [12, 21] and EVs-cargo contents [22, 23] are currently under debate.

Here, we addressed some of these challenges at different levels of analysis. First, we utilized the particle precipitation method, which is time-saving, suited for clinical laboratories, and yielded higher RNA quantities compared to other methods [24]. It precipitated the bulk of circulating miRNA populations, vesicle-trapped and protein-bound [5, 6, 25]. This co-precipitation maximizes the opportunity of permissible disclosure of disease-related markers. Yet, this isolation method is not appropriate to investigate specific miRNA carrier subpopulations. The intensive co-isolation of non-EVs complexes may, for instance, hamper and mask the detection of specific EVs miRNA carriers [26, 27]. Currently, there is an uncertainty in the biological/functional consequences of different particle compartments (EVs- and non-EVs). Also, the mechanism of extracellular RNA (as a “disposal” mechanism of unwanted/debris materials and/or a “control/signaling” mechanism to alter other cells at a distance) remains unclear. Moreover, recent studies identified AGO2 in EVs [23], which has been primarily found in non-EVs-protein complexes, and has further demonstrated its potential function in sorting and secretion of specific miRNAs into EVs, in a KRAS-MEK-dependent manner [26]. These recent findings may suggest that EVs- and non-EVs miRNA carriers are somehow overlapping and cannot be fully regarded as two independent compartments of specific miRNAs, as traditionally thought [5, 6, 28]. Nevertheless, for functional analyses of specific miRNAs other established protocols can be utilized [7, 27].

Secondly, the correlation of miRNA patterns in serum of tumor patients with the corresponding tumor tissues can provide indications of the possible origins of the observed circulating miRNAs. This does not exclude miRNAs that are also present in normal colon tissues or activated by indirect effects of the tumor. Thirdly, only serum samples without hemolysis were considered for analysis to minimize the influence of blood-based miRNA patterns [29]. It is noteworthy that previous studies comparing plasma and serum did not find substantial differences in extracellular miRNA patterns [19]. Yet, differences between blood compartments may arise, e.g. upon platelet activation [19]. Finally, for qPCR-based analysis, utilization of two data normalization methods was deemed more trustworthy.

### Concentration of miRNA carriers improved detection of CRC tissue-related signatures

Our results demonstrated that a simple polymer-based enrichment, prior to sensitive qPCR profiling, substantially improved detection of circulating miRNAs, thereby disclosing twofold relevant CRC-associated candidates that mirror deregulations in corresponding tissues. The recent work of Melo and colleagues [30] also showed that isolation of EVs was necessary to provide a more reliable (Glypican-1-based) diagnostic test for patients with pancreatic cancer, irrespective of the unclear intracellular origin of their marker [31]. Our results are thus consistent with previous reports that highlight circulating EVs as stable [9], degradation-protected and enriched source [32] for miRNA biomarker discovery. The latter sequencing-based study reported 40 miRNAs that were confined inside serum EVs and were not detectable in serum and (PAXgene) blood. Similarly, we found a large set (15/28; >53%) of differentially expressed miRNAs only in patients' particle-concentrated sera (see Supplementary Data). Furthermore, we found that all but one (miR-144) of the identified miRNAs were reported in the Extracellular Vesicles Database (http://microvesicles.org/). Remarkably, many of these candidates, such as miR-15b, miR-21, miR-25, miR-92, miR-93, miR-223 and miR-486, can potentially be used as disease biomarkers in CRC [33].

Our results (Supplementary Tables S8 and S9) also suggest the possibility of crosstalk between these miRNAs and key inflammatory-/cancer-related factors, such as STAT3, and other STATs and cytokines [34]. For instance, miR-21, which was elevated in all CRC sera and tissues, has been reported to be increased in human colon tumors and correlated with tumor stage [35]. When transported by EVs, miR-21 can also induce tumor progression via pro-metastatic inflammatory responses by binding to Toll-like receptors and activation of NF-κB-IL6/TNF-alpha signaling [36]. Another such example is miR-26a, which showed consistent elevation in sera and tissues of metastatic CRC patients (Supplementary Tables S4 and S10). Reported evidence indicated that overexpression of miR-26a can either reduce STAT3 phosphorylation in colon mucosa [37] or suppress tumor growth and metastasis through IL6-STAT3 signaling [38]. Yet, another study showed a reverse effect, where STAT3 can suppress miR-26a expression in T-cell lymphoma [39] (see Supplementary Discussion).

### Insights into the expression patterns of miR-486 and miR-92-a in CRC

In the CRC field, the expression patterns of miR-486 and miR-92a remain under debate. Their expression has been reported to be elevated in metastatic CRC with mutated KRAS, even prior to treatment [40]. Yet, a recent study [15] suggested that the reported elevation of both miRNAs in cancers (including CRC) mainly originated from erythrocytes, with a 20-30 fold increase in case of hemolysis. As hemolysis was absent or minimal in our serum samples, and given the consistent decrease of miR-486 and miR-92a across all particle-concentrated sera (and whole sera in case of miR-486) and corresponding tissues (Table 1, Figure 3, and Supplementary Table S10), our results support the original hypothesis by Pritchard and colleagues [15]. In addition, it was reported [25] that the fluctuations of expression of both miRNAs are likely to rely on the EVs isolation method rather than different levels of AGO2 proteins bound to these miRNAs, as previously suggested [5]. Therefore, the “down-regulation” signature of both miRNAs needs to be validated in larger independent cohorts.

### Evidence of strand-specific differential expression of miR-22 and miR-21 in CRC

Our results suggest a possible “strand-specific” differential expression of extracellular miRNAs in CRC. For instance, both miR-22-3p and miR-22-5p were increased in paired CRC whole- and particle-concentrated sera, with a three-fold higher elevation of miR-22-5p than miR-22-3p in the respective enriched fractions than in whole sera (Table 1; Supplementary Table S3). Yet, a different scenario emerged in tumor tissues, which showed an elevation of miR-22-3p and down-regulation of miR-22-5p. ANOVA analysis (Supplementary Table S7; Figure 3) showed more concordance of expression patterns of miR-22-3p in the matched cancer particle-concentrated sera and tissues. These expression differences suggest an oncogenic role of miR-22-3p and a tumor suppressor function of miR-22-5p. Indeed, more relations to diseases (27 [including CRC] *vs.* two [hepatitis-B, prostate cancer]) and potential target genes (430 *vs.* 186) have been documented for miR-22-3p than of miR-22-5p (miRSearch v3: www.exiqon.com/miRSearch). miR-22 is known as a tumor suppressor in lung [41] and colon cancer [42], but it is unclear which mature form is most relevant. Down-regulation of miR-22 has been shown in CRC tissue and correlated with liver metastasis [43]. Deregulation of miR-22 has also been observed in tissues with IBD [44]. Remarkably, miR-22 is not expressed by blood cells [15]. Altogether, these data suggest that the abundance of miR-22 in the circulation may originate from the diseased (colon) tissue. The second candidate is miR-21. While miR-21-5p was overexpressed in CRC particle-concentrated sera and tissues, miR-21-3p was not detectable (Table 1; Figure 3). Previous studies also showed association of miR-21-5p expression with 134 diseases, including CRC (miRSearch v3).

To our knowledge, few studies have addressed this strand-specific miRNA-expression tendency. Examples are miR-28, miR-125, and miR-34c in CRC [45], lung [46] and cervical cancers [47], respectively. Nevertheless, in agreement with our results, a recent transcriptome analysis revealed a high abundance of both miR-22-3p and miR-21-5p (two of five most overexpressed miRNAs) in a cohort of 88 CRC tumors [48].

### Implication of extracellular miRNAs in CRC and IBD

Out of the identified 14 miRNAs, with consistent patterns of regulation in both neoplastic and inflammatory bowel diseases, the expression trends of both miR-125b-5p and miR-146a-5p were in agreement with our first results. Both of these miRNAs are well known to be implicated in the regulation of immune functions and in CRC and IBD [49–52] (Supplementary Table S9). An additional candidate is miR-30b-5p, which was the only significantly overexpressed miRNA in CRC compared to IBD and the healthy individual serum pools. miR-30b-5p has been reported as a potential prognostic marker that mainly functions as a tumor suppressor [53] and mediator of metastatic and invasion behaviors in CRC [54, 55]. The higher expression of miR-30b in the “vesicle-enriched” CRC sera may indicate that tumor discards undesired inhibitory factors into the circulation via EVs. A higher expression of miR-30b in the circulation of CRC patients, compared to the corresponding CRC tissue and healthy controls (ANOVA, P-value: 0.0022), was also observed in our study (Supplementary Table S7). Taken together, the elevated expression level of miR-30b may suggest its utilization as an indicator for CRC progression.

*In summary*, enrichment of miRNA-containing particles from serum is evolving as a promising strategy for identifying cancer tissue-related miRNA signatures in liquid biopsies. As the clinical relevance and function of most of the known miRNAs are still undetermined, further studies are warranted to explore their specific association with key cellular pathways and inflammation-driven colon carcinogenesis, as well as possible biological implications of strand-specific expression. This may in turn provide insights into the pathogenesis of bowel diseases and lead to more effective therapies.

## MATERIALS AND METHODS

### Subjects

For this study a total number of 71 serum and 25 tissue samples were investigated. In the initial discovery and validation experiments, 29 serum samples, obtained from 19 clinical patients suffering from CRC and from 10 healthy individuals, as well as the corresponding CRC tissue samples (n=25) from the resected tumors were analyzed (details in Supplementary Table S2). The 93 isolated RNAs from these samples were subjected to miRNA qPCR expression profiling (Supplementary Data). For a second independent validation approach, we analysed 42 additional serum samples (seven pools; six samples per pool) of cohorts suffering from CRC (UICC stage II disease; 18 samples in three pools), as well as ulcerative colitis (UC; 18 sera in three pools) and healthy individuals (six sera in one pool) (Supplementary Table S2c). The serum and tissue samples were obtained from the local oncological biomaterial bank of the Comprehensive Cancer Center North, University Clinic Schleswig-Holstein, Campus Kiel, and the Interdisciplinary Center for Biobanking-Lübeck (ICB-L). The sample collection and the analyses in this study were approved by the local review board and all donors gave informed written consent. Sera were prepared by centrifugation (2000 g, 10 min) of blood samples, which had been either collected from healthy donors and patients with UC, by aspiration from the medial cubital vein or were collected from CRC patients prior to surgical tumor resection from a central venous line under general anesthesia. The tumor tissue specimens from patients being operated at the Department of General Surgery, University Clinic Schleswig-Holstein, Campus Kiel and were snap-frozen in liquid nitrogen (n=15) or RNA stabilized by RNAlater reagent (n=10; Qiagen, Hilden, Germany). All samples were stored in the biobanks at minimum −80°C until use.

### Preparation of particle-concentrated and particle-depleted serum fractions

For enrichment of miRNA-containing particles, ExoQuick (SBI/BioCat, Heidelberg, Germany) was used following the manufacturer's instructions. Good performance of this isolation system has been reported previously [25]. To pre-evaluate the appropriate serum volume for particle-enrichment and RNA preparation, isolated particles from different volumes of serum were resuspended in 200 μl Dulbecco's phosphate-buffered saline (PBS; Life Technologies, Darmstadt, Germany) and the total RNA was isolated (Qiagen miRNeasy kit). Subsequent analysis of yield and quality was performed by a Bioanalyzer 2100 (Agilent, Böblingen, Germany) using a RNA6000 Pico-kit (Agilent). Based on these experiments (data not shown), and as a compromise between RNA yield and input volume of clinical sera, 2 ml serum samples were used for enriching the circulating miRNA-containing particles. Thawed sera were pre-cleared by centrifugation at 13.000 xg for 10 min at 4°C and were mixed with 0.5 ml ExoQuick reagent, incubated overnight at 4°C for particle precipitation, and subsequently centrifuged at 1,500 xg for 30 min at 4°C. The pellets (particle-concentrated fractions) were resuspended in 1/10^th^ of the original serum volume, according to the manufacturer's protocol, i.e. in 200 μl PBS. The supernatants were kept as particle-depleted fractions. Nanoparticles tracking analysis (NTA) by a Nanosight NS300 (Malvern GmbH, Herrenberg, Germany) was used to assess size and concentration of isolated particles. Additionally, particle characteristics after isolation by ExoQuick were compared to two other isolation methods and after different storage conditions (Supplementary Figures S6 and S7).

### RNA isolation and quality checks

Total RNA including miRNA was extracted from whole serum, and the resuspended particle-concentrated and particle-depleted sera, using the miRNeasy kit (Qiagen). Briefly, 200 μl of each fraction (unprocessed serum, particle suspension and particle-depleted serum) was homogenized in 1 ml Qiasol (Qiagen) and processed according to the manufacturer's instructions. Similarly, RNA from tissue samples (30-60 mg) was extracted using the same kit after homogenization in Qiasol with a Precellys24 homogenizer and ceramic beads (1.4 mm; PEQLAB, Erlangen, Germany). The RNA was stored at −80°C. Quality controls were undertaken to evaluate pre-analytical and technical imbalances (Supplementary Data).

### cDNA synthesis and qPCR

The RNA samples included in this study were reverse-transcribed into cDNA and run on miRCURY LNA^TM^ RT-miRNA-PCR panels (Exiqon, Vedbaek, Denmark). *The Universal RT miRNA PCR Human panel-I-and-II (v2)*, consisting of 742 miRNA assays, was used in the first screen (30 RNA samples isolated from 10 matched CRC samples as whole sera, particle-concentrated and particle-depleted sera). The second panel, *Universal RT miRNA PCR Custom Pick&Mix*, consisting of 47 miRNA assays, was used for the subsequent experiments with the other 63 RNA samples (Figure 1). Moreover, as a third panel, a *miRCURY LNA^TM^ Universal RT miRNA PCR Human panel I,* which consists of 372 miRNAs, was used to independently analyze the miRNA expression patterns in three entities: CRC, ulcerative colitis (UC), and healthy volunteers (details in Supplementary Table S2c). Three pools of patient's sera for each disease, as well as one serum pool of healthy controls (6 individuals per pool; a total of 42 sera), were investigated. This third miRNA-panel included most of the 45 miRNAs (identified in the initial screen; except for miR-320b, miR-1972, and miR-17-5p) and importantly, all but one (miR-320b) of the 22 miRNAs identified as differentially expressed in the particle-concentrated sera between CRC and healthy individuals (Figure 2). In this additional experiment, all seven serum pools (0.5 ml each) were treated with DNase and RNase prior to isolation of miRNA-containing particles to digest extraneous nucleic acids. Briefly, 0.5 ml pooled sera was mixed with 50 μl 10× DNase-reaction buffer (Promega, Heidelberg, Germany, #M198A), 3 μl DNase (Promega #M6101), and 5 μl RNase A (Sigma-Aldrich, Munich, Germany, #R6148) and incubated for 60 min at RT. Then, 40 μl of stop solution (Promega M199A) and 10 μl (200 units) recombinant RNase inhibitor (ABI/Thermo Fisher) were added. Next, total RNA, including miRNA, from exosomes and other EVs, was isolated using exoRNeasy method (Qiagen) according to the manufacturer's instructions.

### Data filtering and analysis

The amplification efficiencies in the qPCRs were analyzed using algorithms similar to the LinReg software (Supplementary Data). To improve our miRNA-selection, two data normalization methods were independently applied to the raw data of the initial screen (details in Supplementary Data). The overlapping miRNAs from both analyses, which showed statistically significant differential expression between the matched whole and particle-concentrated sera, were considered for follow up and in the combined downstream analyses (Figure 2). To assess miRNA expression differences between samples, fold changes were calculated, as well as, t-tests and multiple testing corrections according to the Benjamini-Hochberg method were applied. Furthermore, the miEAA tool (http://www.ccb.uni-saarland.de/mieaa_tool) was used to perform different overrepresentations and enrichment analyses (Supplementary Data).

## SUPPLEMENTARY MATERIALS AND METHODS, FIGURES AND TABLES

















## Abbreviations

miRNA: microRNA
Cont: control
CRC: colorectal cancer
EVs: extracellular microvesicles
M1: CRC patients with distant metastasis
M0: CRC patients without metastasis
Ser: serum
NTA: Nanoparticle Tracking Analysis
IBD: inflammatory bowel disease
UC: ulcerative colitis
RT: room temperature.
