# Supplementary material for: Concentration of circulating miRNA-containing particles in serum enhances miRNA detection and reflects CRC tissue-related deregulations
Source: Oncotarget. 2016 Sep 23;7(46):75353–65. doi: 10.18632/oncotarget.12205 (PMC5342746; doi:10.18632/oncotarget.12205)
Supplement: Supplementary file 1 [file oncotarget-07-75353-s001.pdf]

# Concentration of circulating miRNA-containing particles in serum enhances miRNA detection and reflects CRC tissue-related deregulations

## SUPPLEMENTARY MATERIALS AND METHODS

### RNA quality checks

To ensure sample quality and quantity, different quality controls were undertaken. To detect the presence of inhibitors, and as a technical control, a UniSp6 RNA spike-in probe (Exiqon, Vedbaek, Denmark) was included in the reverse transcription reaction (RT) to evaluate RT performance variation in all samples. A negative (water) control was co-profiled with the samples to exclude any RNA contamination. Moreover, a sample hemolysis check was performed using the relative expression of two hemolysis indicators, the erythrocyte-specific miRNA-451 and the stable miRNA-23a [1]. Samples showing a hemolysis index below 7.0, calculated using the delta quantification cycle of both miRNAs [$dC_q$(miR-23a - miR-451) ratio], were considered as of good quality, i.e. no or very minimal signs of hemolysis.

### cDNA synthesis and qPCR

The cDNA amplification was performed in a LightCycler® 480 Real-Time PCR System (Roche) in 384 well plates and analyzed using the Roche LC software. The applied qPCR method in the current study has been recently shown to possess a decent efficiency [2].

### Data filtering and analysis

The amplification efficiencies in the real-time PCR experiments were determined by analyzing the amplification curves using algorithms similar to the LinReg software package [3]. The amplification efficiencies in the real-time PCR experiments ranged between 1.8 and 2.1. Individual reactions with efficiencies <1.6 were excluded from the dataset. All assays were examined for distinct melting curves and the Tm's were inspected to be within known specifications for each particular experiment. Any sample crossing point ($C_q$) value, which was not detected with at least 5 $C_q$ cycles less than the corresponding negative control, and with a $C_q < 37$ (upper limit), was considered undetectable. Data that did not pass the quality control criteria were omitted from further analysis.

It is well known that normalization of qPCR miRNA expression data is a persistent challenge and choosing the proper strategy is crucial to obtain reliable results. In the current study, two data normalization methods [4, 5] were independently applied to the same raw data resulting from the initial screen. First, the global mean normalization method, which is based on the average of the assays detected in all samples [4], was applied. This method has been shown to be suitable to normalize qPCR miRNA expression data across different cell types and tissues [6]. The stability of the average of the selected miRNAs for normalization was higher than any single microRNA in the data set as confirmed by the Normfinder software [7]. To further improve our miRNA-selection, the modified global mean normalization strategy, based on the attribution of equal weight to each individual miRNA during normalization [5], was independently applied to the same raw data resulting from the initial screen (Figure 1). Multiple testing correction according to the Benjamini-Hochberg method [8] was applied. P-values <0.05 were considered statistically significant.

To assess miRNA expression differences between samples, fold changes were calculated and t-tests and multiple testing correction according to the Benjamini-Hochberg method were applied. In addition, heat map and unsupervised hierarchical clustering were carried out using the Euclidian distance. ANOVA analysis was also performed to display the expression changes of different miRNAs across all tested samples in five groups. The RNA/cDNA quality controls, miRNA qPCR profiling experiments as well as the statistical analyses for this study were performed at Exiqon services (Vedbaek, Denmark).

Furthermore, the miEAA tool (http://www.ccb.uni-saarland.de/mieaa_tool) was used to perform different overrepresentation and enrichment analyses of the identified 22 miRNAs in the CRC's particle-concentrated serum fractions in different cellular pathways and reported relations to diseases or affected organs/tissues.

## SUPPLEMENTARY RESULTS

### Initial screen: Expression differences between whole sera and particle-concentrated sera

The principal component analysis showed a separation between the matched particle-concentrated and particle-depleted (supernatant) fractions (Supplementary

Figure S2). This was further supported by heat map and unsupervised hierarchical clustering analyses (Supplementary Figure S3).

Each of the two normalizations approaches yielded 44 statistically significant candidates, which showed differential expression between matched whole sera and particle-concentrated fractions. Both methods yielded 43 of 44 of overlapping miRNAs (Supplementary Table S1). For further analysis, the 43 overlapping miRNAs plus the two uniquely identified miRNAs (miR-143; miR-423) in both normalized datasets were considered in the validation experiments.

## Tissue preservation conditions can influence miRNA expression patterns

To assess the effect of different RNA preservation methods for tissue miRNA detection, six of the tested 25 CRC tissue specimens were analyzed as paired samples from the same tissues stored either after snap-freezing in liquid nitrogen or after fixation in RNA-later reagent. A pronounced impact of the preservation method was observed based on the number of the detected miRNAs and on the average $C_q$ values measured (Supplementary Table S5; Supplementary Figure S4). Using a paired t-test, 13 miRNAs were found to be differentially expressed (Supplementary Figure S5). Differential expression of two miRNAs remained significant after correction for multiple testing (Supplementary Table S6). While miR-101-3p was mainly upregulated in tissue samples preserved in liquid nitrogen, miR-15b-5p showed upregulation primarily in tissue samples stored in RNA-later. The reason for this differential expression or the slightly better performance of tissue samples preserved in liquid nitrogen remains unclear. Yet, our results are in agreement with other studies that underscore the general suitability of the routinely processed tissue samples for miRNA detection. Differential expression levels of miRNAs, caused either by different tissue storage strategies [9] or storage time [10] have been reported in similar studies. These observations should be taken into account when interpreting expression data and comparing them with those of other studies. Together with other findings, we considered only the data of the tissues preserved in liquid nitrogen (n=15) in our downstream analyses, in order to avoid additional confounding factors.

## miRNA expression in particle-concentrated serum mirrors deregulation of tumor tissues

Seven of the 22 miRNAs in Table 1 showed inconsistent expression between the particle-concentrated serum fractions and the corresponding tissue samples. This may provide some mechanistic insights on the miRNA release into the extracellular environment by cancer cells. In brief, the expression of five of these seven miRNAs (miR-22-5p, -223-3p, -320b, -335-5p, and -144-3p) were significantly upregulated in the particle-concentrated sera of patients compared to those of the respective controls. The first three of these five miRNAs (miR-22-5p,-223-3p and -320b) did not show any significant deregulation when tissue samples were compared to controls' particle-concentrated fractions. But when the level of these three miRNAs in the patients' particle-concentrated fractions was compared to that of the corresponding tissue samples, a significant downregulation was observed in the tissue samples (P-values, 0.0001, 0.0081 and 0.0030, respectively). The other two miRNAs, miR-335-5p, and -144-3p, were even more downregulated in tissue samples than in the controls' particle-concentrated serum fractions (P-values, 0.0024 and 0.0001, respectively) and in patients' particle-concentrated serum fractions (P-values, $2.48\times10^{-9}$ and $6.45\times10^{-7}$, respectively). The expression of the remaining two miRNAs, let-7d-3p and miR-342-3p, were significantly downregulated in patients' particle-concentrated serum fractions compared to the respective controls (P-values, 0.0007 and 0.0064, respectively), but showed significant upregulation in tissue samples compared to the matched patients' particle-concentrated serum fractions (P-values, 0.0049 and 0.0003, respectively) (Table 1).

## miRNA *in silico* enrichment analyses and relation to cancer and inflammatory pathways

Significant over representation of the identified 22 miRNAs in the particle-concentrated serum fractions have been reported in the context of many diseases, such as "carcinoma" and "adenocarcinoma" (22 and 17 miRNAs; P-values 0.0002 and 0.0002, respectively), "neoplasm_metastasis" (21 miRNAs; P-value, $1.12\times10^{-05}$), "inflammation" (16 miRNAs; P- value 0.0002), and "colonic_neoplasms" (10 miRNAs; P-value, 0.0117). "Organs miRWalk" analysis using the miEAA tool showed significant over representation of our 22 miRNA candidates in different organs or tissues, for instance in "blood serum" (20 miRNAs; P-value, $1.6\times10^{-6}$) and "colon" (15 miRNAs; P-value 0.0002). Moreover, "pathway miRWalk" analysis by the miEAA tool resulted in significant enrichment of most of these 22 miRNAs in inflammation- and cancer-related pathways, such as "apoptosis" (22 miRNAs; P-value, 0.0002), "mTOR" (19 miRNAs; P-value, 0.0002), "RAS" (19 miRNAs; P-value 0.0002), "Inflammation" (20 miRNAs; P-value 0.0002), "TLR" (21 miRNAs; P-value 0.0005), "IL-6" (18 miRNAs; P-value, 0.0006), "STAT3" (14 miRNAs; P-value, 0.0006), "cell cycle" (22 miRNAs; P-value, 0.0009), "P53" (21 miRNAs; P-value, 0.0009), "NF-κB"

(16 miRNAs; P- value, 0.0012), “cytokine-cytokine-receptor-interaction” (21 miRNAs; P-value, 0.0014), “CRC” (22 miRNAs; P-value, 0.0019), “WNT” (21 miRNAs; P-value, 0.0026), and “Jak-STAT-signaling” (20 miRNAs; P-value, 0.0062) (other details in Supplementary Table S8).

## SUPPLEMENTARY DISCUSSION

### Concentration of miRNA-carrying particles improves detection of CRC-related miRNAs

Our observation of a lower detection rate of miRNA in the unprocessed serum compared to the concentrated miRNA-containing particles in corresponding serum seems reasonable based on recent observations and findings. Chevillet and colleagues [11] suggested that miRNA pattern after enrichment of potentially miRNA-rich subpopulations of exosomes may be different from the overall miRNA signature in the same biofluid without enrichment. Moreover, Cheng and colleagues [12] emphasized that the presence of miRNA in cell-free samples is diluted in the plasma and serum volume, and this low abundance can be regarded as a confounding factor in the detection of miRNA by qPCR. Altogether, these observations support the applied strategy in our study, of enriching the “bulk” of miRNA-containing circulating particles (extracellular vesicles and others structures such as RNA-protein and RNA-lipoprotein complexes) as a reasonable approach in the situation of miRNA biomarker discovery. This may also plausibly explain the higher detection rate of miRNA candidates in the concentrated serum fraction compared to the corresponding unprocessed serum.

The number of differentially regulated miRNAs (28 out of 742; ~4%; Figure 2) should be better put into context of other studies. In the present study, following high sensitivity qRT-PCR detection, we applied unique filtering and stringent selection criteria. This strategy resulted in a reliable set of miRNA candidates and is comparable in terms of numbers of regulated transcripts to other studies. For example, the results of a microarray-based analysis followed by a qRT-PCR validation [13], ended up with seven miRNAs that showed significantly higher expression levels in serum EVs of CRC patients (n=88) than in healthy controls (n=11), as well as down-regulation after surgical resection of tumors. Similarly, these seven miRNAs had higher expression levels in colon cancer cell lines (n=5) than in a normal colon-derived cell line. Notably, three of these seven miRNAs, namely miR-21, miR-23a and miR-223, also showed significantly higher expression in our particle-concentrated serum fractions (Table 1). In another study [14], out of 1547 miRNAs profiled using a Universal RT microRNA PCR system (GeneCopoeia, USA) in two pooled tissue samples (28 tumors and 28 paired normal controls), only 93 miRNAs (6%) were firstly identified with significantly dysregulation Colorectal adenocarcinoma relative to normal tissues. Further filtering and downstream analyses ended up with only 32 miRNAs (2.1%) that were able to distinguish cancer tissues from normal tissues, as well as to identify well- and moderately differentiated cancers. Remarkably, a realistic subset of these 32 miRNAs, for example miR-23b, miR-125b, miR-26a, let-7i, miR-99a, and miR-100, were also highlighted in our study as potential biomarker candidates (Figures 2 and 4). In a third study [12], which utilized a different EVs isolation kit system (Norgen Biotek, Canada) followed by next-generation sequencing, 15 and 40 miRNAs were detected (out of 386 and 412 miRNAs; 4% and 10%, respectively) to be confined inside serum- and plasma-EVs, respectively. Those 15 and 40 miRNAs were not detectable in whole (PAXgene) blood. Similarly, in our study 15 of 28 (>50%) miRNAs were found to be only deregulated in patients' particle-concentrated sera (Figure 2).

### Concentration of miRNA-carrying particles improved detection of CRC-related signatures

Examples of miRNAs with consistent upregulation in particle-concentrated CRC sera and tissues, but not in whole sera, are miR-125 and miR-23a. While miR-125 is known as an important prognostic indicator for CRC [15], miR-23a is a critical regulator of CRC migration and metastasis [16].

## SUPPLEMENTARY FOOTNOTE TO TABLE 1

In each comparison presented in Table 1, two respective columns are shown, one for the fold changes and the other for the adjusted/corrected P-values. When the fold change has a positive value, this means that the first item in the respective comparison showed higher miRNA expression level than the second item of the same comparison; and when the P-value does not indicate significance, implies that the compared expression levels are statistically the same (i.e., similar expression).

In the first (I) and third (III) comparison, it is clear that the first 10 miRNAs showed higher expression (fold changes with positive values and significant P-values $\leq$ 0.05) in the particle-concentrated CRC serum samples and tumor tissues compared to particle-concentrated sera of the controls, respectively. Yet, in the second comparison (II. Particle-Concentrated Sera (CRC) vs. Tissue (CRC)), seven miRNAs are described by negative fold changes (printed in italics in Table 1), which reflect lower expression levels of these miRNAs in particle-concentrated CRC sera than in CRC tissue samples. The corresponding P-values of the remaining three miRNAs, although having positive fold changes,

indicated non-significance, hence their expression levels were statistically not different. Based on this evaluation, we concluded that the first 10 miRNAs in Table 1 showed consistent expression levels in sera and tissues of the investigated CRC cases.

The same holds true for the five down-regulated miRNAs (miR-486-5p, -93-5p, -92a-3p, -146a-5p, and -221-3p). Here, in the first (I) and third (III) comparison, it is clear that these 5 miRNAs showed lower expression in the particle-concentrated CRC serum samples and CRC tissues samples compared to the particle-concentrated serum samples of the controls (fold changes with negative values and P-values ≤ 0.05). Yet, in the second comparison, two of these miRNAs with barely negative fold change showed P-values not reaching significance. Therefore, these expression levels (of miR-92a-3p, -221-3p) are rated as equal. This was also concluded for miR-93-5p and miR-146a-5p, which also had non-significant (positive) fold-changes. But remarkably, miR-486-5p showed the highest downregulation in the CRC tissues compared to the corresponding sera with high significance (P-value, $1.14\times10^{-14}$).

Accordingly, a total of 15 out of the 22 miRNA candidates (68%) showed consistent expression patterns in the sera and tissues of CRC samples. Moreover, the ANOVA results, across all the tested samples (Supplementary Table S7), clearly support the afore-mentioned interpretation. Taking miR-21-5p as an example, the average normalized $C_q$ values ($dC_q$) were higher in the CRC tissues than in the particle-concentrated CRC sera (0.8377 vs. -0.28552), which point to higher expression in the tumor than in the corresponding particle-concentrated sera, which is in agreement with our interpretation of the results presented in Table 1.

## REFERENCES


1. Blondal T, Jensby Nielsen S, Baker A, Andreasen D, Mouritzen P, Wrang Teilum M and Dahlsveen IK. Assessing sample and miRNA profile quality in serum and plasma or other biofluids. Methods. 2013; 59:S1-6.
2. Mestdagh P, Hartmann N, Baeriswyl L, Andreasen D, Bernard N, Chen C, Cheo D, D'Andrade P, DeMayo M, Dennis L, Derveaux S, Feng Y, Fulmer-Smentek S, et al. Evaluation of quantitative miRNA expression platforms in the microRNA quality control (miRQC) study. Nat Methods. 2014; 11:809-815.
3. Ruijter JM, Ramakers C, Hoogaars WM, Karlen Y, Bakker O, van den Hoff MJ and Moorman AF. Amplification efficiency: linking baseline and bias in the analysis of quantitative PCR data. Nucleic Acids Res. 2009; 37:e45.
4. Mestdagh P, Van Vlierberghe P, De Weer A, Muth D, Westermann F, Speleman F and Vandesompele J. A novel and universal method for microRNA RT-qPCR data normalization. Genome Biol. 2009; 10:R64.
5. D'Haene B, Mestdagh P, Hellemans J and Vandesompele J. miRNA expression profiling: from reference genes to global mean normalization. Methods Mol Biol. 2012; 822:261-272.
6. Wang WX, Danaher RJ, Miller CS, Berger JR, Nubia VG, Wilfred BS, Neltner JH, Norris CM and Nelson PT. Expression of miR-15/107 Family MicroRNAs in Human Tissues and Cultured Rat Brain Cells. Genomics Proteomics Bioinformatics. 2014; 12:19-30.
7. Andersen CL, Jensen JL and Orntoft TF. Normalization of real-time quantitative reverse transcription-PCR data: a model-based variance estimation approach to identify genes suited for normalization, applied to bladder and colon cancer data sets. Cancer Res. 2004; 64:5245-5250.
8. Benjamini Y, Drai D, Elmer G, Kafkafi N and Golani I. Controlling the false discovery rate in behavior genetics research. Behav Brain Res. 2001; 125:279-284.
9. Li J, Smyth P, Flavin R, Cahill S, Denning K, Aherne S, Guenther SM, O'Leary JJ and Sheils O. Comparison of miRNA expression patterns using total RNA extracted from matched samples of formalin-fixed paraffin-embedded (FFPE) cells and snap frozen cells. BMC Biotechnol. 2007; 7:36.
10. Siebolts U, Varnholt H, Drebber U, Dienes HP, Wickenhauser C and Odenthal M. Tissues from routine pathology archives are suitable for microRNA analyses by quantitative PCR. J Clin Pathol. 2009; 62:84-88.
11. Chevillet JR, Kang Q, Ruf IK, Briggs HA, Vojtech LN, Hughes SM, Cheng HH, Arroyo JD, Meredith EK, Gallichotte EN, Pogosova-Agadjanyan EL, Morrissey C, Stirewalt DL, et al. Quantitative and stoichiometric analysis of the microRNA content of exosomes. Proc Natl Acad Sci U S A. 2014; 111:14888-14893.
12. Cheng L, Sharples RA, Scicluna BJ and Hill AF. Exosomes provide a protective and enriched source of miRNA for biomarker profiling compared to intracellular and cell-free blood. J Extracell Vesicles. 2014; 3.
13. Ogata-Kawata H, Izumiya M, Kurioka D, Honma Y, Yamada Y, Furuta K, Gunji T, Ohta H, Okamoto H, Sonoda H, Watanabe M, Nakagama H, Yokota J, Kohno T and Tsuchiya N. Circulating exosomal microRNAs as biomarkers of colon cancer. PLoS One. 2014; 9:e92921.
14. Wu X, Xu X, Li S, Wu S, Chen R, Jiang Q, Liu H, Sun Y, Li Y and Xu Y. Identification and validation of potential biomarkers for the detection of dysregulated microRNA by qPCR in patients with colorectal adenocarcinoma. PLoS One. 2015; 10:e0120024.
15. Nishida N, Yokobori T, Mimori K, Sudo T, Tanaka F, Shibata K, Ishii H, Doki Y, Kuwano H and Mori M. MicroRNA miR-125b is a prognostic marker in human colorectal cancer. Int J Oncol. 2011; 38:1437-1443.

16. Jahid S, Sun J, Edwards RA, Dizon D, Panarelli NC, Milsom JW, Sikandar SS, Gumus ZH and Lipkin SM. miR-23a promotes the transition from indolent to invasive colorectal cancer. Cancer Discov. 2012; 2:540-553.
17. Iliopoulos D, Jaeger SA, Hirsch HA, Bulyk ML and Struhl K. STAT3 activation of miR-21 and miR-181b-1 via PTEN and CYLD are part of the epigenetic switch linking inflammation to cancer. Mol Cell. 2010; 39:493-506.
18. Rozovski U, Calin GA, Setoyama T, D’Abundo L, Harris DM, Li P, Liu Z, Grgurevic S, Ferrajoli A, Faderl S, Burger JA, O’Brien S, Wierda WG, Keating MJ and Estrov Z. Signal transducer and activator of transcription (STAT)-3 regulates microRNA gene expression in chronic lymphocytic leukemia cells. Mol Cancer. 2013; 12:50.
19. Zhang J, Xiao Z, Lai D, Sun J, He C, Chu Z, Ye H, Chen S and Wang J. miR-21, miR-17 and miR-19a induced by phosphatase of regenerating liver-3 promote the proliferation and metastasis of colon cancer. Br J Cancer. 2012; 107:352-359.
20. Schmitt MJ, Philippidou D, Reinsbach SE, Margue C, Wienecke-Baldacchino A, Nashan D, Behrmann I and Kreis S. Interferon-gamma-induced activation of Signal Transducer and Activator of Transcription 1 (STAT1) up-regulates the tumor suppressing microRNA-29 family in melanoma cells. Cell Commun Signal. 2012; 10:41.
21. Koukos G, Polytarchou C, Kaplan JL, Morley-Fletcher A, Gras-Miralles B, Kokkotou E, Baril-Dore M, Pothoulakis C, Winter HS and Iliopoulos D. MicroRNA-124 regulates STAT3 expression and is down-regulated in colon tissues of pediatric patients with ulcerative colitis. Gastroenterology. 2013; 145:842-852 e842.
22. Wang B, Hsu SH, Frankel W, Ghoshal K and Jacob ST. Stat3-mediated activation of microRNA-23a suppresses gluconeogenesis in hepatocellular carcinoma by down-regulating glucose-6-phosphatase and peroxisome proliferator-activated receptor gamma, coactivator 1 alpha. Hepatology. 2012; 56:186-197.
23. Hu R and O’Connell RM. MiR-23b is a safeguard against autoimmunity. Nat Med. 2012; 18:1009-1010.
24. Cai Y, Chen H, Jin L, You Y and Shen J. STAT3-dependent transactivation of miRNA genes following Toxoplasma gondii infection in macrophage. Parasit Vectors. 2013; 6:356.
25. Hatziapostolou M, Polytarchou C, Aggelidou E, Drakaki A, Poultsides GA, Jaeger SA, Ogata H, Karin M, Struhl K, Hadzopoulou-Cladaras M and Iliopoulos D. An HNF4alpha-miRNA inflammatory feedback circuit regulates hepatocellular oncogenesis. Cell. 2011; 147:1233-1247.
26. Iliopoulos D, Hirsch HA and Struhl K. An epigenetic switch involving NF-kappaB, Lin28, Let-7 MicroRNA, and IL6 links inflammation to cell transformation. Cell. 2009; 139:693-706.
27. Wang X, Cao L, Wang Y, Liu N and You Y. Regulation of let-7 and its target oncogenes (Review). Oncol Lett. 2012; 3:955-960.
28. Luo X, Zhang L, Li M, Zhang W, Leng X, Zhang F, Zhao Y and Zeng X. The role of miR-125b in T lymphocytes in the pathogenesis of systemic lupus erythematosus. Clin Exp Rheumatol. 2013; 31:263-271.
29. Liu LH, Li H, Li JP, Zhong H, Zhang HC, Chen J and Xiao T. miR-125b suppresses the proliferation and migration of osteosarcoma cells through down-regulation of STAT3. Biochem Biophys Res Commun. 2011; 416:31-38.
30. Parisi C, Arisi I, D’Ambrosi N, Storti AE, Brandi R, D’Onofrio M and Volonte C. Dysregulated microRNAs in amyotrophic lateral sclerosis microglia modulate genes linked to neuroinflammation. Cell Death Dis. 2013; 4:e959.
31. Chen Q, Wang H, Liu Y, Song Y, Lai L, Han Q, Cao X and Wang Q. Inducible microRNA-223 down-regulation promotes TLR-triggered IL-6 and IL-1beta production in macrophages by targeting STAT3. PLoS One. 2012; 7:e42971.
32. Haneklaus M, Gerlic M, O'Neill LA and Masters SL. miR-223: infection, inflammation and cancer. J Intern Med. 2013; 274:215-226.
33. Zhu L, Chen L, Shi CM, Xu GF, Xu LL, Zhu LL, Guo XR, Ni Y, Cui Y and Ji C. MiR-335, an adipogenesis-related microRNA, is involved in adipose tissue inflammation. Cell Biochem Biophys. 2014; 68:283-290.
34. Liu S, Patel SH, Ginestier C, Ibarra I, Martin-Trevino R, Bai S, McDermott SP, Shang L, Ke J, Ou SJ, Heath A, Zhang KJ, Korkaya H, et al. MicroRNA93 regulates proliferation and differentiation of normal and malignant breast stem cells. PLoS Genet. 2012; 8:e1002751.
35. Foshay KM and Gallicano GI. miR-17 family miRNAs are expressed during early mammalian development and regulate stem cell differentiation. Dev Biol. 2009; 326:431-443.
36. Bhaumik D, Scott GK, Schokrpur S, Patil CK, Orjalo AV, Rodier F, Lithgow GJ and Campisi J. MicroRNAs miR-146a/b negatively modulate the senescence-associated inflammatory mediators IL-6 and IL-8. Aging (Albany NY). 2009; 1:402-411.
37. Bhaumik D, Scott GK, Schokrpur S, Patil CK, Campisi J and Benz CC. Expression of microRNA-146 suppresses NF-kappaB activity with reduction of metastatic potential in breast cancer cells. Oncogene. 2008; 27:5643-5647.
38. Saki N, Abroun S, Soleimani M, Mortazavi Y, Kaviani S and Arefian E. The roles of miR-146a in the differentiation of Jurkat T-lymphoblasts. Hematology. 2014; 19:141-147.
39. Hwang MS, Yu N, Stinson SY, Yue P, Newman RJ, Allan BB and Dornan D. miR-221/222 targets adiponectin receptor 1 to promote the epithelial-to-mesenchymal transition in breast cancer. PLoS One. 2013; 8:e66502.

40. Asquith M, Pasala S, Engelmann F, Haberthur K, Meyer C, Park B, Grant KA and Messaoudi I. Chronic ethanol consumption modulates growth factor release, mucosal cytokine production, and microRNA expression in nonhuman primates. Alcohol Clin Exp Res. 2014; 38:980-993.
41. Zhang C, Han L, Zhang A, Yang W, Zhou X, Pu P, Du Y, Zeng H and Kang C. Global changes of mRNA expression reveals an increased activity of the interferon-induced signal transducer and activator of transcription (STAT) pathway by repression of miR-221/222 in glioblastoma U251 cells. Int J Oncol. 2010; 36:1503-1512.
42. Yang X, Liang L, Zhang XF, Jia HL, Qin Y, Zhu XC, Gao XM, Qiao P, Zheng Y, Sheng YY, Wei JW, Zhou HJ, Ren N, Ye QH, Dong QZ and Qin LX. MicroRNA-26a suppresses tumor growth and metastasis of human hepatocellular carcinoma by targeting interleukin-6-Stat3 pathway. Hepatology. 2013; 58:158-170.
43. Zhu H, Vishwamitra D, Curry CV, Manshouri R, Diao L, Khan A and Amin HM. NPM-ALK up-regulates iNOS expression through a STAT3/microRNA-26a-dependent mechanism. J Pathol. 2013; 230:82-94.

## SUPPLEMENTARY FIGURES AND TABLES

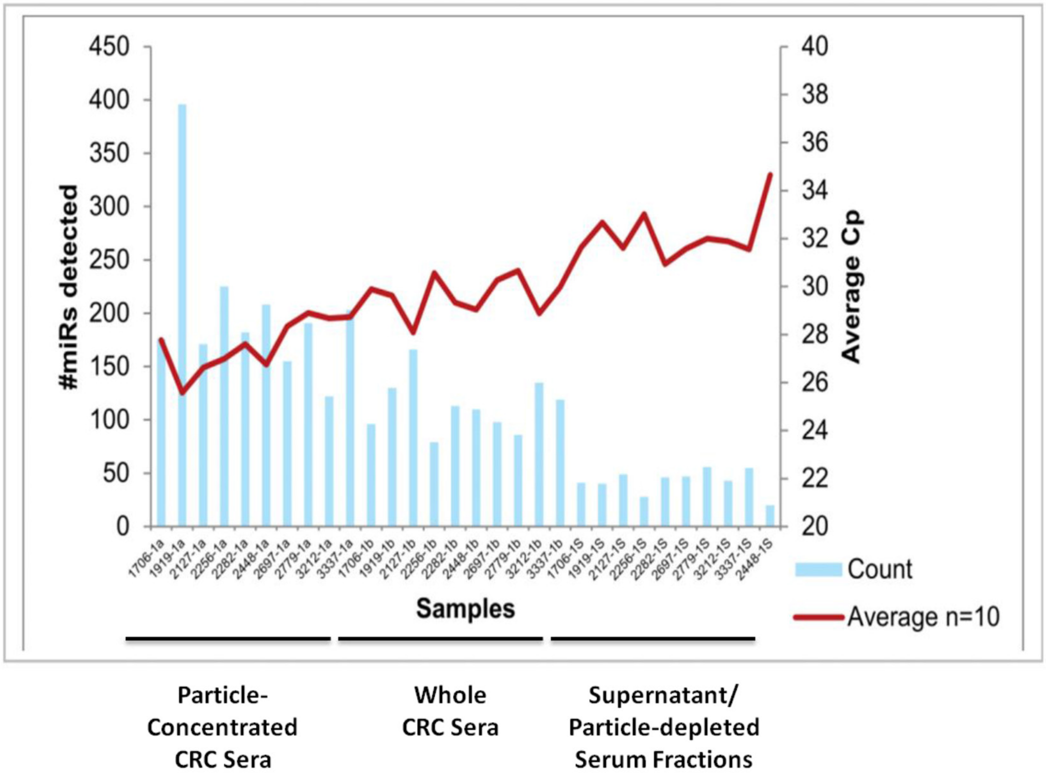


**Supplementary Figure S1: Initial screen – The number of the detected miRNAs in the tested three serum compartments (whole [b], particle-concentrated [a], and particle-depleted[s]).** This graphical illustration shows the number of miRNA detected in the initial screen, using qPCR of 742 miRNA panel, in matched samples (whole serum, particle-concentrated sera, and particle-depleted sera (supernatant)), of 10 CRC patients with metastasis (all at tumor stage IV). More miRNAs were detected in particle-concentrated fractions than in corresponding unprocessed serum and particle-depleted samples. The observation that a considerable number of detected miRNAs were picked up in the particle-depleted serum samples may imply that the applied polymer-based precipitation method needs further improvements in order to enable better concentration of different miRNA forms and miRNA-containing particles in serum samples. Moreover, although these results clearly underscore higher detection rate of circulating miRNAs in the particle-concentrated serum fractions, we can't rule out the influence of the larger volume of the sera used in the concentration step compared to the unprocessed serum. Moreover, qPCR data may be influenced by a threshold cDNA input. To deal with these confounding factors, we applied two different data normalization methods and didn't pay so much attention to the differences in the number of detected miRNAs in the initial screening stage. Rather, in the downstream analyses, after the validation experiments, we focused on comparing the differentially deregulated miRNAs in matched serum fractions of the patient and respective healthy control samples (Figures 2 and 3) and monitoring their expression in the paired tissue samples (Table 1 and Supplementary Tables S7 and S10).

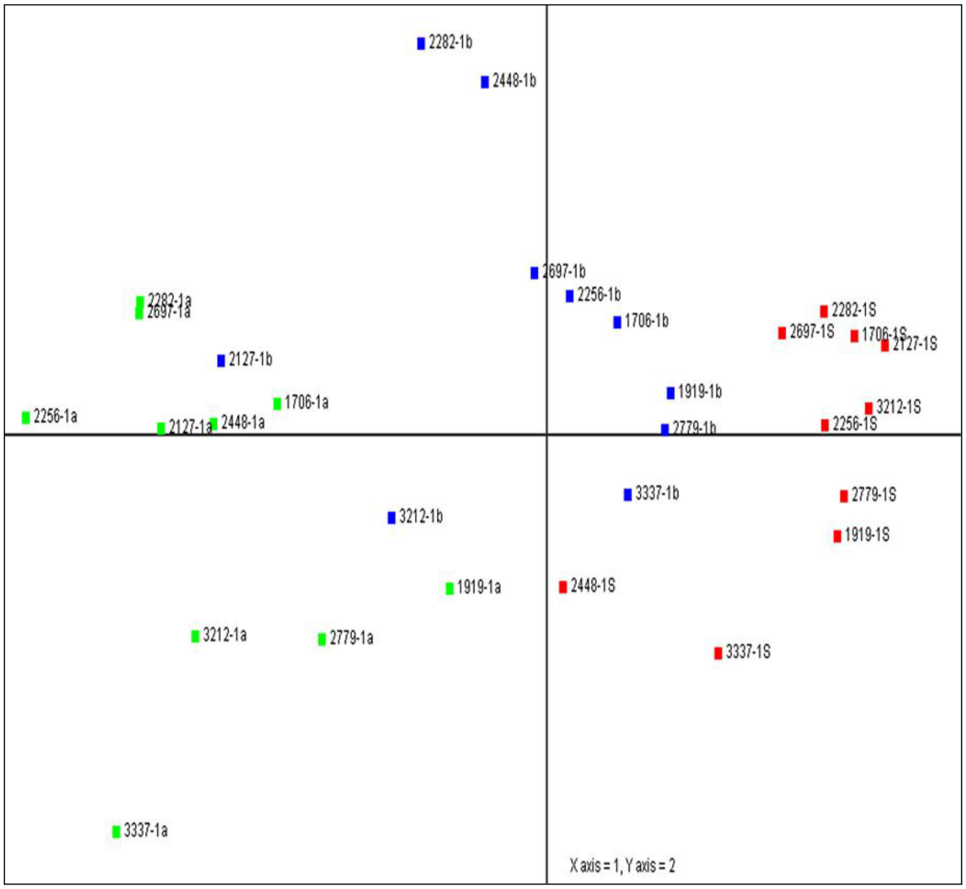


**Supplementary Figure S2: Initial screen – Principal component analysis (PCA) plot.** The principal component analysis is performed on all samples, and on the 30 miRNAs most differentially expressed. The normalized ($dC_q$) values have been used for the analysis. The sample source separates relatively well on the first primary component but they also seem to somewhat pair on the second component (green = particle-concentrated serum samples; blue = whole serum and red= supernatant or particle-depleted serum samples). Here, the particle-concentrated serum samples (green boxes) are clearly separated from the particle-depleted serum (supernatant) samples (red boxes) and the whole sera (blue boxes) in the middle.

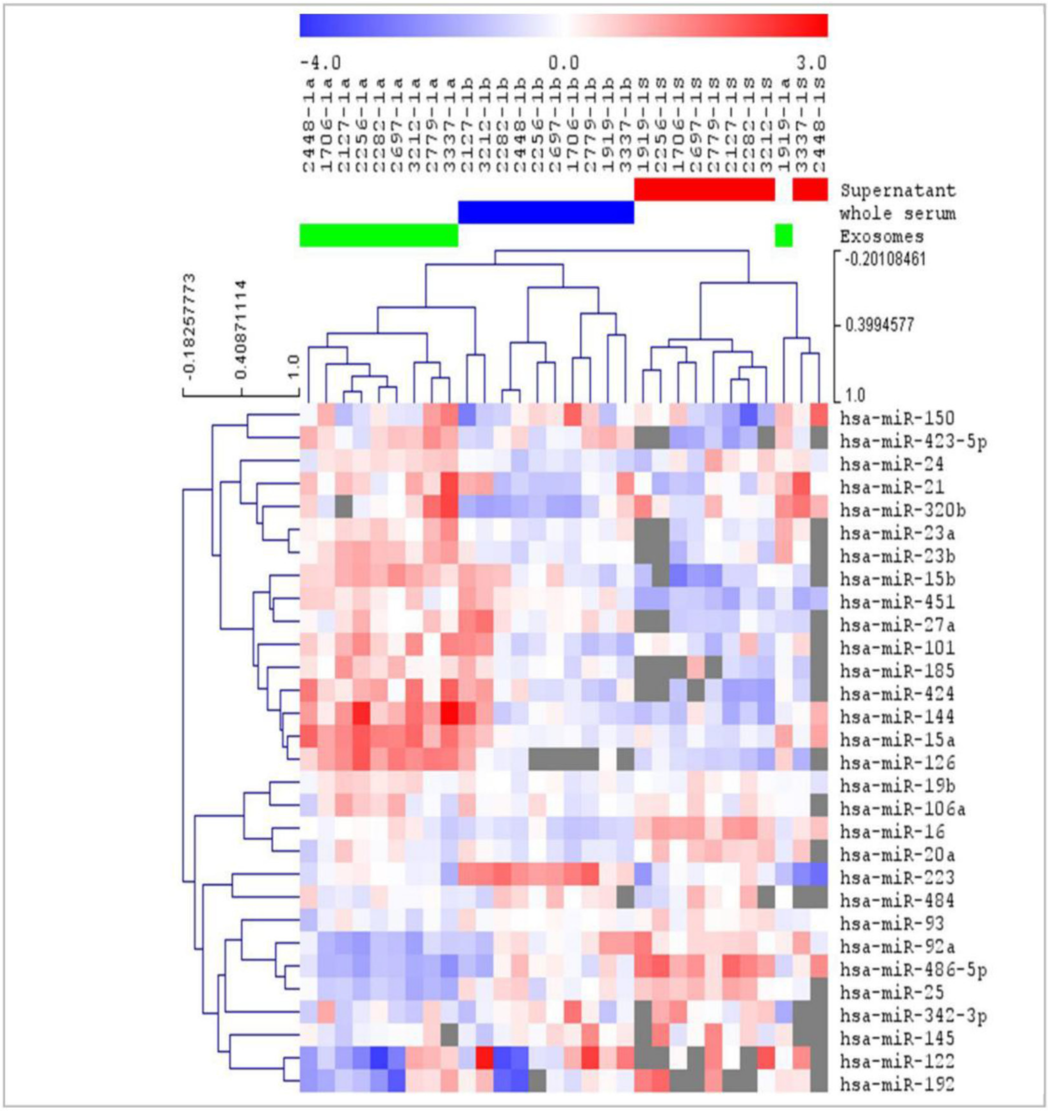


**Supplementary Figure S3: Initial screen – Heat map and unsupervised hierarchical clustering of the samples tested.** The heat map diagram shows the result of the two-way hierarchical clustering of miRNAs and samples. Each row represents one miRNA and each column represents one sample. The miRNA clustering tree is shown on the left. The color scale shown at the top illustrates the relative expression level of a miRNA across all samples: red color represents an expression level above mean, blue color represents expression lower than the mean and grey color represents missing data. The clustering is performed on all samples, and on the 30 most differentially expressed miRNAs (note that missing data are more common in the supernatant samples). The normalized ($dC_q$) values have been used for the analysis. A clear differential expression of the tested miRNAs emerged among different serum fractions investigated. (Serum exosomes in this figure refers to particle-concentrated serum samples).

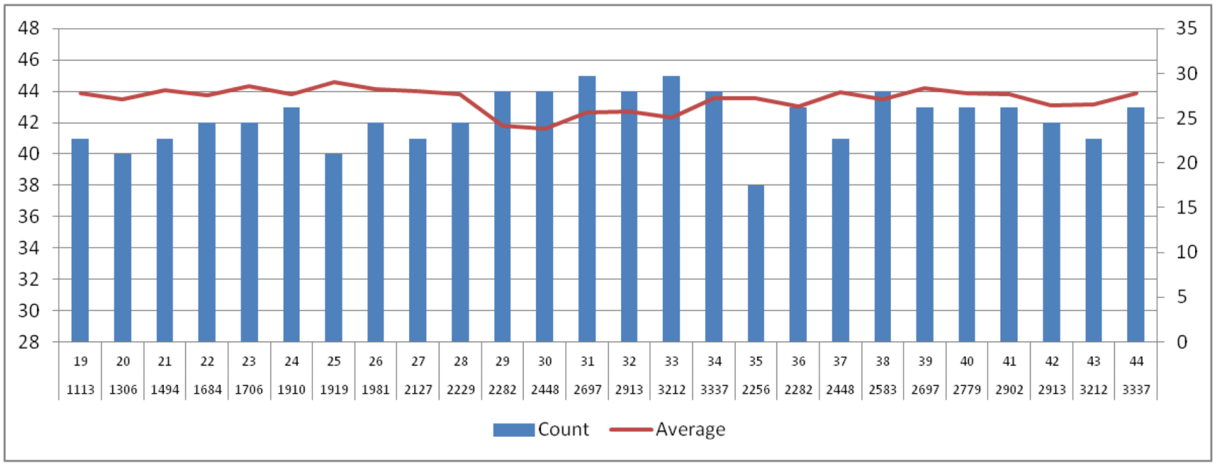


**Supplementary Figure S4: miRNAs detected in all tested CRC tissue samples (paired, n=25).** On average, 42 microRNA were detected per sample. A general high quality and quantity of RNAs were isolated from all tissue samples. Expression profiling of the selected 45 miRNAs, using the custom qPCR panel, showed a general good performance of all the 25 tissue samples tested as shown on this Figure: an average of 42 of the tested 45 miRNAs were detected in all tested tissue samples.

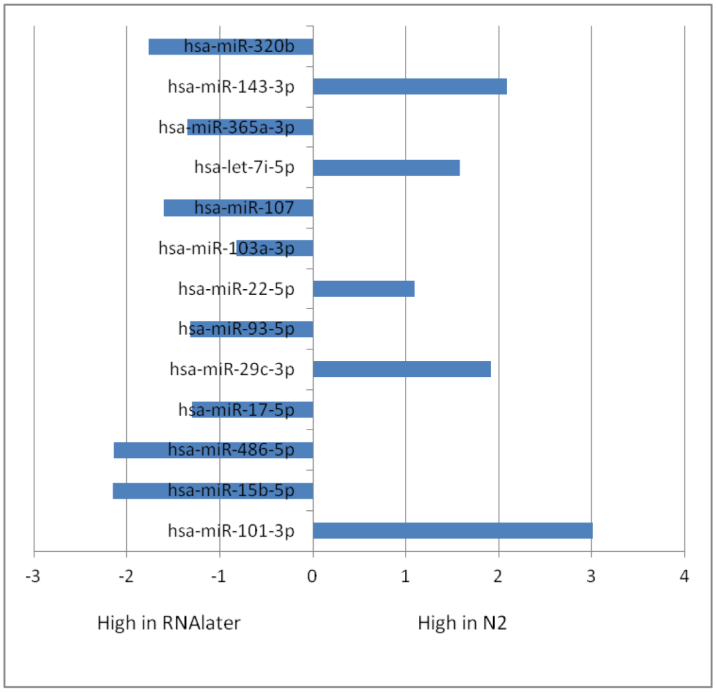


**Supplementary Figure S5: Differentially expressed miRNAs across CRC tissue samples.** When comparing the two tissue storage conditions (snap-frozen in liquid nitrogen or RNA-later reagent), using a paired t-test, 13 miRNAs were found to differentially expressed using a cutoff of P-value < 0.05.

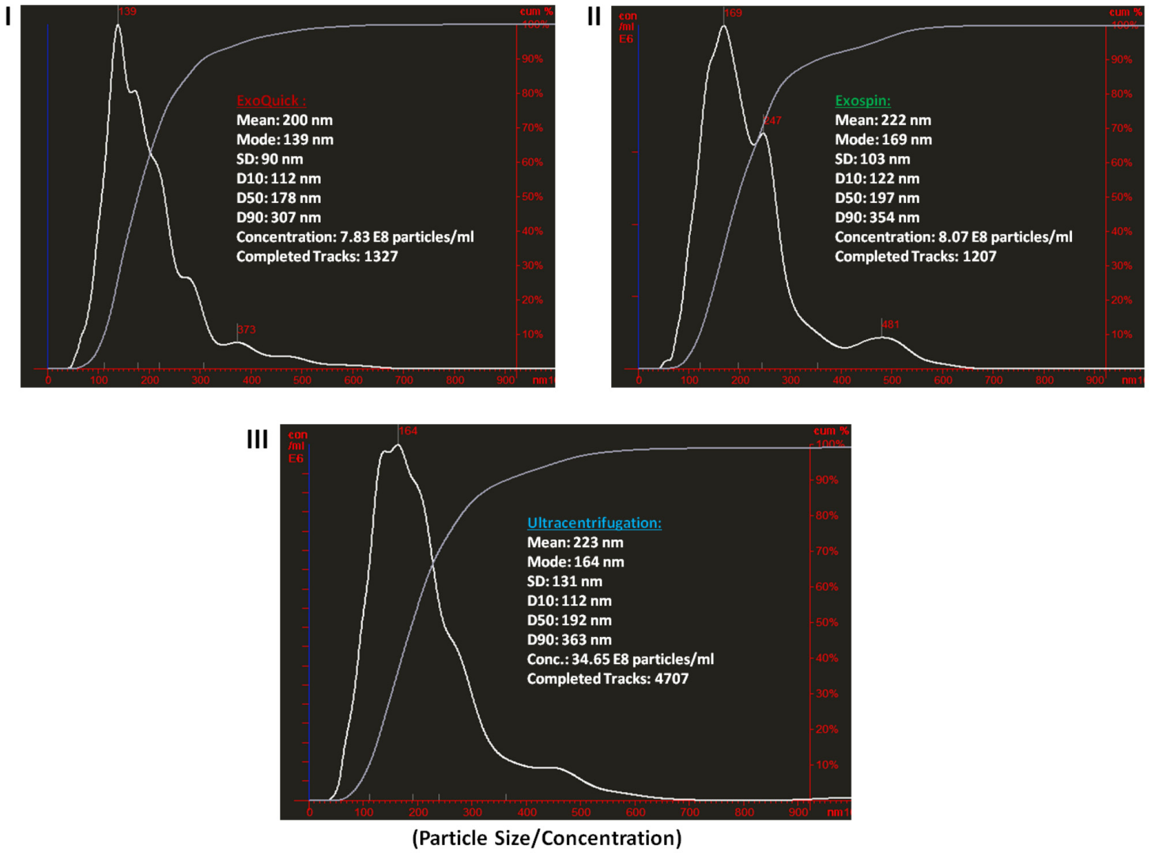


**Supplementary Figure S6: Comparison of particle isolation methods using Nanoparticle Tracking Analysis (NTA).** miRNA-containing particles were isolated (from serum free cancer cell culture supernatant; 1:10 dilution) by three different methods, namely (I) ExoQuick precipitation (SBI/BioCat, Heidelberg, Germany), (II) CellGS Exospin (Cell Guidance Systems, Cambridge, UK), and (III) the ultracentrifugation procedure. The results of NTA measurement (NanoSight NS300; software version 2.3) were compared regarding the particle size. The mean sizes in all three measurements were 200 nm, 222 nm and 223 nm, respectively. The concentration of miRNA-carrying particles obtained using ultracentrifugation protocol was higher, than that of the kit-base systems (ExoQuick and ExoSpin). This might be due to the higher volume (twofold of the input material) used in the ultracentrifugation experiments to get a comparable visible particle-precipitated pellet.

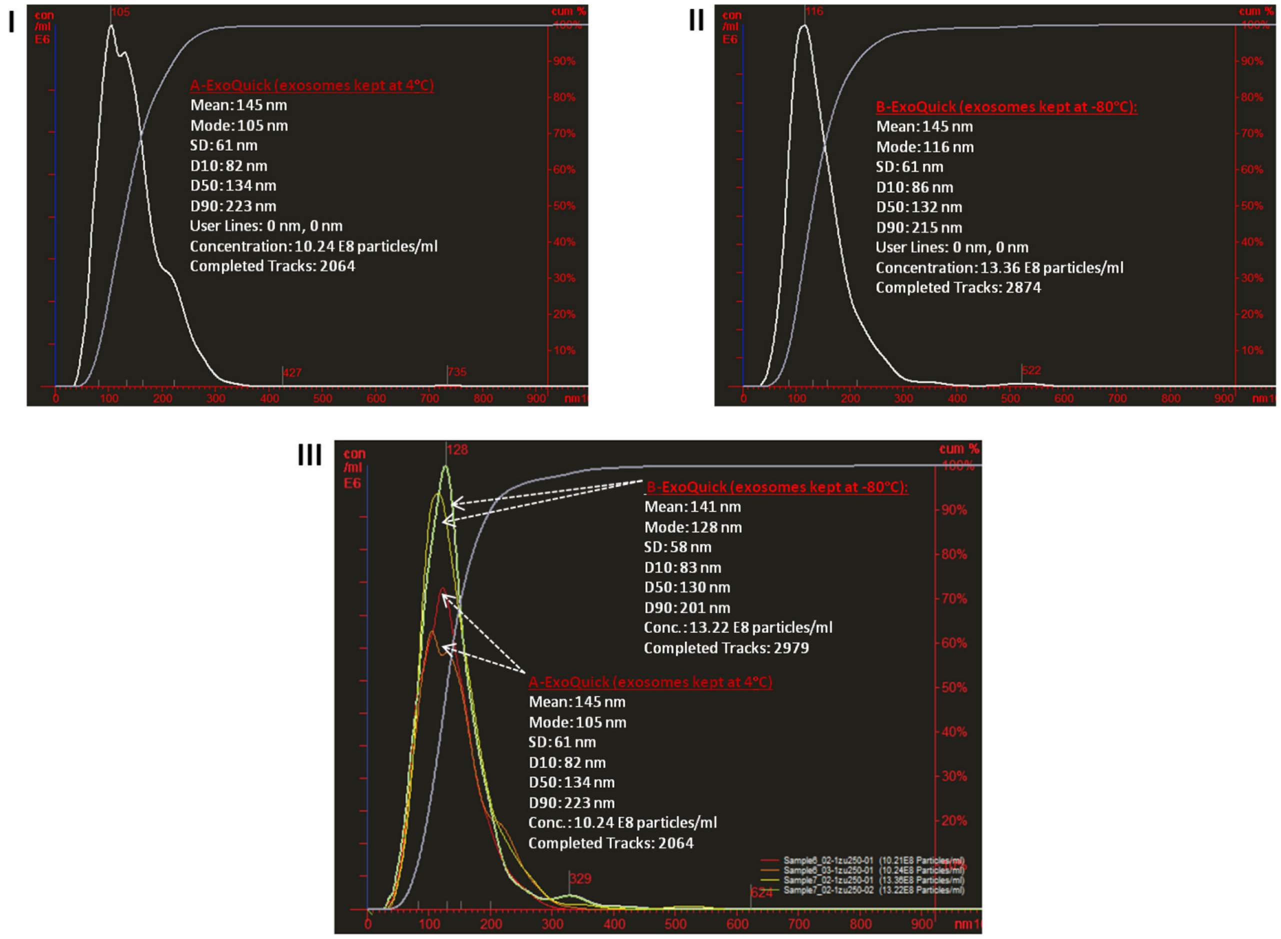


(Particle Size/Concentration)

**Supplementary Figure S7: Effect of storage conditions on isolated particle's size, concentration and reproducibility of NTA analysis.** Two particle-concentrated serum samples (1:250 dilution) were enriched from patients' sera by ExoQuick precipitation. While isolated particles from one sample A (I) were kept at 4ºC, particles from sample B (II) were stored at -80ºC. NTA analyses (NanoSight NS300; software version 2.3) shows that particle size mean was 145 nm in both cases (I and II). A slight difference in particle concentration was noticed. To check for the reproducibility of the NTA measurement, each sample was measured at two different time points. As shown in the lower Figure III, the NTA measurement was reproducible in both samples A and B. It is noticeable that, although in each independent analysis (shown in Supplementary Figure S6 and S7) a peak of particle size close to 100-150 nm has been observed, differences in mean particle sizes (and heterogeneous peak shoulders) have been observed in different preparations, even using the same particle isolation method. This can be affected by different factors, such as different sample sources (serum free cell culture supernatant vs. serum samples, in the analyses shown in Supplementary Figures S6 and S7, respectively), sample dilutions (1:250 vs. 1:10, respectively), ratio of protein aggregates in the preparation, and respective heterogeneity of (co)isolated particles from different samples.

**Supplementary Table S1: Expression differences and fold changes of detected miRNAs of matched whole serum and particle-concentrated serum samples of CRC patients (initial screen).**

**See Supplementary File 1**

**Supplementary Table S2: Samples tested.**

**See Supplementary File 2**

**Supplementary Table S3: Differentially expressed miRNAs between particle-concentrated serum of CRC and control samples**

| Rank | miRNA | A (Average $dC_q$ of particle-concentrated sera of 11 metastatic CRC patients) | C (Average $dC_q$ of particle-concentrated sera of 8 non-metastatic CRC patients) | F (Average $dC_q$ particle-concentrated sera of 10 healthy controls) | AC-F ($ddC_q$; difference between patients and controls) | P-value | Benjamini-hochberg corrected P-value |
|---|---|---|---|---|---|---|---|
| 1 | **hsa-miR-22-3p** | -2.504 | -4.229 | -5.670 | 2.673 | $8.23\times10^{-07}$ | $3.04\times10^{-05}$ |
| 2 | **hsa-miR-21-5p** | 0.497 | -4.199 | -5.051 | 4.766 | $4.34\times10^{-06}$ | $5.65\times10^{-05}$ |
| 3 | **hsa-miR-29c-3p** | -2.608 | -4.652 | -5.432 | 2.482 | $4.58\times10^{-06}$ | $5.65\times10^{-05}$ |
| 4 | **hsa-miR-22-5p** | 2.426 | -4.307 | -5.948 | 6.130 | $8.00\times10^{-06}$ | $7.40\times10^{-05}$ |
| 5 | **hsa-miR-101-3p** | -1.183 | -3.814 | -5.270 | 3.334 | $1.20\times10^{-05}$ | $8.91\times10^{-05}$ |
| 6 | **hsa-miR-486-5p** | -0.585 | 2.0886 | 2.801 | -2.119 | $2.09\times10^{-05}$ | 0.0001 |
| 7 | hsa-miR-23a-3p | 0.195 | -0.176 | -0.653 | 0.673 | $4.16\times10^{-05}$ | 0.0002 |
| 8 | hsa-miR-335-5p | -3.419 | -3.369 | -5.229 | 1.832 | $4.52\times10^{-05}$ | 0.0002 |
| 9 | hsa-miR-93-5p | -0.752 | 0.455 | 0.771 | -0.951 | $9.25\times10^{-05}$ | 0.0003 |
| 10 | **hsa-miR-23b-3p** | -1.985 | -3.671 | -3.939 | 1.259 | 0.0001 | 0.0004 |
| 11 | **hsa-miR-144-3p** | 0.313 | -3.316 | -4.378 | 3.330 | 0.0001 | 0.0004 |
| 12 | hsa-let-7d-3p | -5.020 | -2.291 | -2.104 | -2.006 | 0.0002 | 0.0007 |
| 13 | **hsa-miR-423-5p** | -1.269 | -4.962 | -5.017 | 2.516 | 0.0003 | 0.00109 |
| 14 | hsa-miR-92a-3p | 1.125 | 0.757 | 2.077 | -1.126 | 0.0010 | 0.0026 |
| 15 | **hsa-miR-24-3p** | 0.879 | -1.783 | -1.691 | 1.473 | 0.0011 | 0.0029 |
| 16 | hsa-miR-146a-5p | -4.655 | -3.411 | -3.324 | -0.777 | 0.0013 | 0.0030 |
| 17 | **hsa-miR-223-3p** | 2.426 | -2.102 | -1.738 | 2.151 | 0.0023 | 0.0051 |
| 18 | hsa-miR-221-3p | -3.291 | -0.352 | -0.659 | -1.325 | 0.0032 | 0.0064 |
| 19 | hsa-miR-342-3p | -3.753 | -2.026 | -1.729 | -1.205 | 0.0033 | 0.0064 |
| 20 | **hsa-let-7f-5p** | -4.323 | -3.500 | -5.577 | 1.328 | 0.0051 | 0.0095 |
| 21 | hsa-miR-320b | 1.019 | 1.003 | 0.216 | 0.795 | 0.0076 | 0.0134 |
| 22 | hsa-miR-125b-5p | -4.806 | -3.731 | -5.464 | 1.243 | 0.0269 | 0.0454 |

miRNAs in bold prints (n=12) represent those (overlapping) miRNAs that were also detected to be differentially expressed in the whole serum CRC samples (see Supplementary Table S4 and Figure 2). The remaining miRNAs (n=10) are the ones that were detected to be differentially expressed in particle-concentrated CRC samples only (and not in the matched whole serum CRC samples). Positive $ddC_q$ values indicate upregulation and negative values indicate downregulation.

**Supplementary Table S4: Differentially expressed miRNAs between whole serum of CRC and control samples**

| Rank | miRNA | B (Average $dC_q$ of whole sera of 11 metastatic CRC patients) | D (Average $dC_q$ of whole sera of 8 non metastatic CRC patients) | G (Whole sera of 10 healthy controls) | BD-G ($ddC_q$; difference between patients and controls) | P-value | Benjamini-hochberg corrected P-value |
|---|---|---|---|---|---|---|---|
| 1 | **hsa-miR-21-5p** | -0.021 | ND | -5.419 | 5.397 | $2.71\times10^{-09}$ | $1.05\times10^{-07}$ |
| 2 | **hsa-miR-423-5p** | -1.464 | -3.060 | -5.843 | 4.010 | $1.19\times10^{-08}$ | $2.33\times10^{-07}$ |
| 3 | **hsa-miR-144-3p** | -0.674 | -1.594 | -3.609 | 2.628 | $1.54\times10^{-06}$ | $2.01\times10^{-05}$ |
| 4 | **hsa-miR-101-3p** | -1.823 | -2.207 | -4.058 | 2.1073 | $1.05\times10^{-05}$ | 0.0001 |
| 5 | **hsa-miR-24-3p** | 0.414 | -1.381 | -1.931 | 1.672 | $2.26\times10^{-05}$ | 0.0001 |
| 6 | hsa-miR-26a-5p | -2.355 | -2.246 | -5.133 | 2.798 | $4.09\times10^{-05}$ | 0.0002 |
| 7 | **hsa-miR-29c-3p** | -3.062 | -3.218 | -5.093 | 2.016 | $5.50\times10^{-05}$ | 0.0003 |
| 8 | **hsa-miR-22-3p** | -4.193 | -3.908 | -5.216 | 1.051 | $9.14\times10^{-05}$ | 0.0004 |
| 9 | **hsa-miR-223-3p** | 4.346 | -0.213 | -0.528 | 2.848 | 0.0002 | 0.0010 |
| 10 | **hsa-miR-22-5p** | 4.346 | 1.052 | -3.464 | 6.454 | 0.0003 | 0.0013 |
| 11 | **hsa-miR-486-5p** | 0.871 | 2.446 | 2.773 | -1.155 | 0.0031 | 0.0110 |
| 12 | **hsa-let-7f-5p** | -4.980 | ND | -6.338 | 1.358 | 0.0058 | 0.0189 |
| 13 | **hsa-miR-23b-3p** | -2.629 | -2.836 | -3.904 | 1.205 | 0.0064 | 0.0193 |

miRNAs in bold prints (n=12) represent those (overlapping) miRNAs that were also detected to be differentially expressed in particle-concentrated samples (see Supplementary Table S3 and Figure 2).

**Supplementary Table S5: Difference in miRNA content and qPCR average $C_q$ values between CRC tissue samples (snap-frozen and stored liquid nitrogen) to the matched samples (stored in RNA-later reagent (n=6))**

| | Average (snap-frozen liquid nitrogen) | Average (RNA-later reagent) | StDev | P-value |
|---|---|---|---|---|
| miRNAs detected | 44.3 | 42.2 | 1.35 | 0.0061 |
| Average $C_q$ | 25.26 | 27.25 | 1.46 | 0.0147 |

The above table lists the average number of assays detected in the CRC tissue samples provided in replicates either snap-frozen in liquid N2 or stored in RNA-later. As shown in this Table, more miRNAs were detected in tissue samples stored in snap-frozen liquid nitrogen than those stored in RNA-later (average 44.3 vs. 42.2 miRNAs, respectively; standard deviation: 1.35, P-value of the t-test: 0.0061). The same applies to the $C_q$ values of the qPCR reactions, which were 25.26 and 27.25 for samples stored in liquid nitrogen and RNA-later, respectively (standard deviation 1.46; P value 0.0147). Both t-tests thus indicated that there were significant differences between the number and $C_q$ values of the expressed miRNAs in tissue samples stored in different conditions.

**Supplementary Table S6: Differentially expressed miRNAs between matched CRC tissue samples, snap-frozen in liquid nitrogen (n=6) or stored in RNA-later reagent (n=6)**

| Name | Snap-frozen liquid N2 (average $dC_q$) | RNA-later solution (average $dC_q$) | $ddC_q$ | P-value | Benjamini-hochberg corrected P-value |
|---|---|---|---|---|---|
| **hsa-miR-101-3p** | **0.47** | **-2.54** | **3.011** | **0.0010** | **0.0291** |
| **hsa-miR-15b-5p** | **-0.99** | **1.16** | **-2.153** | **0.0016** | **0.0291** |
| hsa-miR-486-5p | -6.50 | -4.36 | -2.135 | 0.0057 | 0.0702 |
| hsa-miR-17-5p | -2.30 | -1.01 | -1.297 | 0.0087 | 0.0801 |
| hsa-miR-29c-3p | 1.61 | -0.31 | 1.914 | 0.0131 | 0.0832 |
| hsa-miR-93-5p | 0.32 | 1.64 | -1.317 | 0.0166 | 0.0832 |
| hsa-miR-22-5p | -3.03 | -4.13 | 1.096 | 0.0186 | 0.0832 |
| hsa-miR-103a-3p | 1.42 | 2.25 | -0.822 | 0.0200 | 0.0832 |
| hsa-miR-107 | 0.15 | 1.75 | -1.604 | 0.0202 | 0.0832 |
| hsa-let-7i-5p | 0.48 | -1.10 | 1.581 | 0.0277 | 0.1026 |

Table showing microRNA names, average $dC_q$ values, followed by $ddC_q$ and p-values from a paired test between the two groups.
Shown in this Table the top 10 miRNAs that were differentially expressed between matched tissue samples sanp-frozen in liquid nitrogen and RNA-later reagent.
Two miRNAs, namely, miR-101-3p qand miR-15b-5p, remained with significantly P-values (marked in bold) after multiple testing corrections.

**Supplementary Table S7: The results of the ANOVA analysis across all the tested samples.**

**See Supplementary File 3**

**Supplementary Table S8: Selected results from the miEAA analysis tool of the identified 22 miRNAs in the particle-concentrated CRC Sera.**

**See Supplementary File 4**

**Supplementary Table S9: Possible interplay of the identified 22 miRNAs in the particle-concentrated sera with key inflammatory and cancer-related pathways.**

**See Supplementary File 5**

**Supplementary Table S10: Particle-concentrated serum miRNA patterns of CRC patients with metastasis reflect tissue-related expression.**

**See Supplementary File 6**

**Supplementary Table S11: Possible interplay of the identified 22 miRNAs in the particle-concentrated sera of metastatic CRC patients with key inflammatory and cancer-related pathways.**

**See Supplementary File 7**
