# Supplementary material for: Concentration of circulating miRNA-containing particles in serum enhances miRNA detection and reflects CRC tissue-related deregulations
Source: Oncotarget. 2016 Sep 23;7(46):75353–65. doi: 10.18632/oncotarget.12205 (PMC5342746; doi:10.18632/oncotarget.12205)
Supplement: Supplementary file 2 [file oncotarget-07-75353-s002.docx]

**Supplemental Table S1: Expression Differences and Fold Changes of Detected miRNAs of Matched Whole Serum and Particle-Concentrated Serum Samples of CRC Patients (Initial Screen)**

|  | | | **First Normalization I**  **[4]** | | |  | **Bonferroni=** | **0.0006** |  |  | | | **Second Normalization**  **[5]** | | |  | **Bonferroni=** | **0.0006** |
| --- | --- | --- | --- | --- | --- | --- | --- | --- | --- | --- | --- | --- | --- | --- | --- | --- | --- | --- |
| **Order** | **miRNA** | **Count** | **Stdev** | **Average dC_q_ Pellet** | **Average dC_q_ whole sera** | **Difference (logFC)** | **Fold Change** | **P-value** |  | **Order** | **miRNA** | **Count** | **Stdev** | **Average dC_q_ Pellet** | **Average dC_q_ whole sera** | **Difference (logFC)** | **Fold Change** | **P-value** |
| 1 | hsa-miR-23b | 20 | 0.50245 | -0.968 | -1.786 | 0.8182 | 1.7632 | 1.54×10^-08^ |  | 6 | hsa-miR-23b | 20 | 0.5700 | -2.344 | -3.28049 | 0.9363 | 1.9137 | 8.83×10^-06^ |
| 2 | hsa-miR-22 | 19 | 1.0715 | -1.486 | -3.338 | 1.8523 | 3.6108 | 1.31×10^-07^ |  | 4 | hsa-miR-22 | 19 | 1.1444 | -2.862 | -4.836 | 1.9744 | 3.9298 | 8.22×10^-07^ |
| 3 | hsa-miR-103 | 20 | 0.7989 | 1.281 | 0.291 | 0.9906 | 1.9870 | 6.12×10^-07^ |  | 1 | hsa-miR-103 | 20 | 0.9485 | -0.094 | -1.202 | 1.1087 | 2.1566 | 1.42×10^-07^ |
| 4 | hsa-miR-223 | 20 | 1.0288 | 3.443 | 5.190 | -1.7462 | -3.3548 | 9.45×10^-07^ |  | 7 | hsa-miR-223 | 20 | 1.0885 | 2.067 | 3.696 | -1.6280 | -3.0910 | 2.98×10^-05^ |
| 5 | hsa-miR-15a | 20 | 0.7671 | 2.515 | 1.212 | 1.3029 | 2.4673 | 1.79×10^-06^ |  | 5 | hsa-miR-15a | 20 | 0.8565 | 1.139 | -0.281 | 1.4210 | 2.6779 | 1.75×10^-06^ |
| 6 | hsa-miR-25 | 20 | 0.8183 | -3.189 | -2.017 | -1.1722 | -2.2537 | 4.38×10^-06^ |  | 11 | hsa-miR-25 | 20 | 0.7506 | -4.565 | -3.511 | -1.0541 | -2.0765 | 1.64×10^-04^ |
| 7 | hsa-miR-107 | 20 | 0.7776 | 0.392 | -0.541 | 0.9335 | 1.9099 | 4.73×10^-06^ |  | 2 | hsa-miR-107 | 20 | 0.9253 | -0.983 | -2.035 | 1.0516 | 2.0729 | 2.12×10^-07^ |
| 8 | hsa-miR-24 | 20 | 0.4086 | 1.896 | 1.257 | 0.6392 | 1.5575 | 1.19×10^-05^ |  | 3 | hsa-miR-24 | 20 | 0.5036 | 0.520 | -0.236 | 0.7573 | 1.6904 | 7.66×10^-07^ |
| 9 | hsa-miR-23a | 20 | 0.4725 | 1.213 | 0.637 | 0.5764 | 1.4911 | 3.91×10^-05^ |  | 9 | hsa-miR-23a | 20 | 0.4811 | -0.162 | -0.856 | 0.6945 | 1.6184 | 6.38×10^-05^ |
| 10 | hsa-miR-130a | 16 | 1.1569 | -2.304 | -4.488 | 2.1837 | 4.5434 | 4.66×10^-05^ |  | 12 | hsa-miR-130a | 16 | 1.2719 | -3.680 | -5.961 | 2.2814 | 4.8618 | 1.78×10^-04^ |
| 11 | hsa-miR-1974 | 19 | 1.8740 | 1.470 | 4.328 | -2.8582 | -7.2514 | 5.38×10^-05^ |  | 10 | hsa-miR-1974 | 19 | 1.8863 | 0.057 | 2.834 | -2.7767 | -6.8532 | 8.52×10^-05^ |
| 12 | hsa-miR-92a | 20 | 1.0620 | 2.142 | 3.459 | -1.3162 | -2.4902 | 8.27×10^-05^ |  | 17 | hsa-miR-92a | 20 | 0.9396 | 0.766 | 1.965 | -1.1981 | -2.2944 | 6.64×10^-04^ |
| 13 | hsa-miR-320b | 19 | 1.2072 | 2.034 | 0.453 | 1.5805 | 2.9909 | 8.52×10^-05^ |  | 8 | hsa-miR-320b | 19 | 1.1523 | 0.621 | -1.040 | 1.6620 | 3.1647 | 4.87×10^-05^ |
| 14 | hsa-miR-26a | 19 | 0.9243 | -0.286 | -1.500 | 1.2147 | 2.3211 | 2.03×10^-04^ |  | 14 | hsa-miR-26a | 19 | 1.1003 | -1.661 | -2.998 | 1.3369 | 2.5261 | 2.75×10^-04^ |
| 15 | hsa-miR-1979 | 20 | 1.3312 | 0.104 | 2.029 | -1.9249 | -3.7973 | 2.08×10^-04^ |  | 15 | hsa-miR-1979 | 20 | 1.2834 | -1.271 | 0.535 | -1.8068 | -3.4987 | 3.35×10^-04^ |
| 16 | hsa-let-7i | 20 | 0.5910 | -3.469 | -2.612 | -0.8575 | -1.8119 | 3.62×10^-04^ |  | 28 | hsa-let-7i | 20 | 0.6231 | -4.845 | -4.106 | -0.7393 | -1.6695 | 4.22×10^-03^ |
| 17 | hsa-miR-221 | 20 | 0.7496 | -2.273 | -1.110 | -1.1630 | -2.2393 | 5.64×10^-04^ |  | 22 | hsa-miR-221 | 20 | 0.7196 | -3.649 | -2.604 | -1.0449 | -2.0632 | 2.19×10^-03^ |
| 18 | hsa-miR-486-5p | 20 | 0.9881 | 0.432 | 1.714 | -1.2827 | -2.4330 | 6.76×10^-04^ |  | 23 | hsa-miR-486-5p | 20 | 0.9175 | -0.943 | 0.220 | -1.1646 | -2.2417 | 2.27×10^-03^ |
| 19 | hsa-miR-21 | 20 | 0.8853 | 1.514 | 0.822 | 0.6927 | 1.6164 | 1.16×10^-03^ |  | 21 | hsa-miR-21 | 20 | 0.8995 | 0.138 | -0.671 | 0.8109 | 1.7543 | 2.19×10^-03^ |
| 20 | hsa-miR-15b | 20 | 0.5310 | 0.321 | -0.181 | 0.5028 | 1.4170 | 1.22×10^-03^ |  | 16 | hsa-miR-15b | 20 | 0.7037 | -1.054 | -1.675 | 0.6209 | 1.5379 | 6.33×10^-04^ |
| 21 | hsa-miR-424 | 20 | 0.8309 | -0.277 | -1.041 | 0.7644 | 1.6988 | 1.29×10^-03^ |  | 13 | hsa-miR-424 | 20 | 0.8777 | -1.653 | -2.535 | 0.8826 | 1.8437 | 2.48×10^-04^ |
| 22 | hsa-miR-93 | 20 | 0.4112 | 0.265 | 0.850 | -0.5847 | -1.4998 | 1.92×10^-03^ |  | 37 | hsa-miR-93 | 20 | 0.4539 | -1.110 | -0.643 | -0.4666 | -1.3819 | 1.73×10^-02^ |
| 23 | hsa-miR-151-5p | 19 | 0.9375 | -1.810 | -3.104 | 1.2942 | 2.4524 | 2.57×10^-03^ |  | 19 | hsa-miR-151-5p | 19 | 1.0234 | -3.186 | -4.612 | 1.4260 | 2.6871 | 1.47×10^-03^ |
| 24 | hsa-miR-30b | 20 | 0.6881 | -1.534 | -2.132 | 0.5981 | 1.5138 | 2.61×10^-03^ |  | 18 | hsa-miR-30b | 20 | 0.8274 | -2.910 | -3.626 | 0.7163 | 1.6429 | 7.66×10^-04^ |
| 25 | hsa-miR-122 | 20 | 1.9043 | -1.336 | -0.167 | -1.1689 | -2.2484 | 3.49×10^-03^ |  | 35 | hsa-miR-122 | 20 | 1.8719 | -2.712 | -1.661 | -1.0507 | -2.0716 | 1.11×10^-02^ |
| 26 | hsa-miR-146a | 16 | 0.6360 | -3.6382 | -4.396 | 0.7581 | 1.6913 | 3.58×10^-03^ |  | 32 | hsa-miR-146a | 16 | 0.7216 | -5.014 | -5.842 | 0.8286 | 1.7760 | 8.17×10^-03^ |
| 27 | hsa-miR-32 | 20 | 0.9297 | -1.789 | -2.840 | 1.0511 | 2.0722 | 5.59×10^-03^ |  | 27 | hsa-miR-32 | 20 | 1.0043 | -3.165 | -4.334 | 1.1693 | 2.2491 | 3.91×10^-03^ |
| 28 | hsa-miR-335 | 18 | 1.0417 | -2.401 | -3.738 | 1.3368 | 2.5261 | 8.28×10^-03^ |  | 29 | hsa-miR-335 | 18 | 1.0904 | -3.777 | -5.268 | 1.4906 | 2.8102 | 4.64×10^-03^ |
| 29 | hsa-miR-144 | 20 | 1.0153 | 1.330 | 0.168 | 1.1621 | 2.2379 | 8.57×10^-03^ |  | 25 | hsa-miR-144 | 20 | 1.1042 | -0.044 | -1.325 | 1.2803 | 2.4289 | 2.58×10^-03^ |
| 30 | hsa-let-7d* | 15 | 1.4802 | 0.315 | -0.967 | 1.2829 | 2.4334 | 9.41×10^-03^ |  | 31 | hsa-let-7d* | 15 | 1.3521 | -1.063 | -2.517 | 1.4546 | 2.7409 | 7.12×10^-03^ |
| 31 | hsa-miR-101 | 20 | 0.7904 | -0.166 | -0.979 | 0.8133 | 1.7572 | 9.51×10^-03^ |  | 20 | hsa-miR-101 | 20 | 0.8726 | -1.542 | -2.473 | 0.9314 | 1.9071 | 1.50×10^-03^ |
| 32 | hsa-miR-29c | 20 | 0.7569 | -1.591 | -2.219 | 0.62828 | 1.5457 | 9.61×10^-03^ |  | 26 | hsa-miR-29c | 20 | 0.7916 | -2.96 | -3.713 | 0.7464 | 1.6776 | 3.22×10^-03^ |
| 33 | hsa-miR-22* | 19 | 0.7321 | -4.589 | -3.816 | -0.7734 | -1.7093 | 9.79×10^-03^ |  | 40 | hsa-miR-22* | 19 | 0.6812 | -5.965 | -5.314 | -0.6512 | -1.5706 | 2.95×10^-02^ |
| 34 | hsa-let-7f | 17 | 1.0222 | -3.306 | -4.144 | 0.8379 | 1.7875 | 1.05×10^-02^ |  | 34 | hsa-let-7f | 17 | 1.1745 | -4.682 | -5.581 | 0.8991 | 1.8649 | 1.04×10^-02^ |
| 35 | hsa-miR-342-3p | 20 | 0.8951 | -2.736 | -2.137 | -0.5984 | -1.5141 | 1.28×10^-02^ |  | 44 | hsa-miR-342-3p | 20 | 0.9511 | -4.112 | -3.631 | -0.4803 | -1.3951 | 4.34×10^-02^ |
| 36 | hsa-miR-126 | 15 | 0.6333 | 1.881 | 0.787 | 1.0942 | 2.1351 | 1.53×10^-02^ |  | 30 | hsa-miR-126 | 15 | 0.8096 | 0.505 | -0.823 | 1.3292 | 2.5128 | 6.07×10^-03^ |
| 37 | hsa-miR-27b | 19 | 1.1178 | -1.105 | -2.149 | 1.0441 | 2.0621 | 1.72×10^-02^ |  | 33 | hsa-miR-27b | 19 | 1.0672 | -2.490 | -3.643 | 1.1528 | 2.2236 | 1.01×10^-02^ |
| 38 | hsa-miR-125b | 15 | 1.0106 | -3.856 | -2.882 | -0.9744 | -1.9649 | 2.16×10^-02^ |  | 41 | hsa-miR-125b | 15 | 0.9517 | -5.121 | -4.376 | -0.7453 | -1.6764 | 3.43×10^-02^ |
| 39 | hsa-miR-17 | 18 | 0.6062 | -3.425 | -4.116 | 0.6913 | 1.6147 | 2.26×10^-02^ |  | 38 | hsa-miR-17 | 18 | 0.7964 | -4.757 | -5.619 | 0.8612 | 1.8166 | 2.01×10^-02^ |
| 40 | hsa-miR-185 | 20 | 0.3867 | -0.754 | -1.080 | 0.3265 | 1.2540 | 2.29×10^-02^ |  | 39 | hsa-miR-185 | 20 | 0.5465 | -2.130 | -2.574 | 0.4447 | 1.3610 | 2.14×10^-02^ |
| 41 | hsa-miR-365 | 19 | 1.0789 | -4.583 | -3.459 | -1.1241 | -2.1797 | 2.29×10^-02^ |  | 42 | hsa-miR-365 | 19 | 0.9709 | -5.959 | -4.957 | -1.0020 | -2.0028 | 3.48×10^-02^ |
| 42 | hsa-let-7d | 17 | 0.7852 | -4.003 | -4.567 | 0.5643 | 1.4787 | 3.88×10^-02^ |  | 36 | hsa-let-7d | 17 | 0.9539 | -5.379 | -6.122 | 0.7432 | 1.6739 | 1.50×10^-02^ |
| 43 | hsa-miR-99a | 19 | 0.9497 | -3.204 | -3.714 | 0.5090 | 1.4232 | 4.10×10^-02^ |  | 24 | hsa-miR-99a | 19 | 0.8787 | -4.580 | -5.212 | 0.6312 | 1.5489 | 2.48×10^-03^ |
| 44 | hsa-miR-143 | 20 | 0.5540 | -3.222 | -2.887 | -0.3349 | -1.2613 | 4.60×10^-02^ |  | - | - | - | - | - | - | - | - | - |
| - | - | - | - | - | - | - | - | - |  | 43 | hsa-miR-423-5p | 20 | 0.6596 | -1.627 | -2.115 | 0.4877 | 1.4022 | 3.95×10^-02^ |

Data in this Table reordered according to the results of the first normalization [4]. Applying two independent normlization methods (D'Haene, et al., 2012; Mestdagh, et al., 2009), as described in the Materials and Methods section, resulted in 44 statistically significant miRNA candiates from each method, which showed differential expression between matched whole serum and particle-concentrated serum samples. Forty-three out of these 44 miRNAs (~98%) were overlapping, i.e. detected in both methods. The uniquely identified miRNAs from each normalization method were miR-143 and miR-423, in the first and second normalization methods, respectively. All the 45 miRNAs (43 overlapping and two uniquely identified) were subjected to second screen/validation.

hsa: abbreviation in the miRNA nomenclature that stands for human miRNA and is derived from *homo* *sapiens*

Pellet: refers to particle-concentrated sera

Adjusted P-value: t-test P-value after adjustment for multiple testing

Stdev: standard deviation

dC_q_: delta C_q_ is the C_q_ value for each primer pair for each sample normalized to the global mean of that sample.

logFC: lof fold change (is the difference between the “pellet (=particle-concentrated serum fraction)” and “serum (unprocessed whole serum)”

FC: Fold change
