# Supplementary material for: Concentration of circulating miRNA-containing particles in serum enhances miRNA detection and reflects CRC tissue-related deregulations
Source: Oncotarget. 2016 Sep 23;7(46):75353–65. doi: 10.18632/oncotarget.12205 (PMC5342746; doi:10.18632/oncotarget.12205)
Supplement: Supplementary file 3 [file oncotarget-07-75353-s003.docx]

**Supplemental Table S2: Samples Tested**

**Supplemental Table S2a: Patients’ Serum Samples Tested in the Initial Screen**

| **Sample no.** | **Type** | **Serum fractions tested*** | ***Tissue Preservation** | **Age** | **Gender** | **UICC Stage** | **pM** | **pN** | **pT** | **Grade** | **Organ** | **Radi-cality** | **Neo-adjuvant treatment** | **Metastasis** | **Months survival post primary surgery** |
| --- | --- | --- | --- | --- | --- | --- | --- | --- | --- | --- | --- | --- | --- | --- | --- |
| 1 | CRC | whole, particle-concentrated &  particle-depleted | Liquid N2 & RNA-later | 64 | m | IV | M1 | N2 | T3 | **G3** | rectum | R2 | no | lung | 13 |
| 2 | CRC | whole, particle-concentrated &  particle-depleted | Liquid N2 & RNA-later | 61 | f | IV | M1 | N2 | T3 | G2 | sigmoid colon | R2 | no | lung, liver | 25.5 |
| 3 | CRC | whole, particle-concentrated &  particle-depleted | Liquid N2 & RNA-later | 70 | m | IV | M1 | N0 | T3 | G2 | sigmoid colon | R2 | no | lung, liver | 28 |
| 4 | CRC | whole, particle-concentrated &  particle-depleted | *RNA-later only* | 68 | f | IV | M1 | N2 | T3 | G2 | colon | R0 | no | liver | 16.5 |
| 5 | CRC | whole, particle-concentrated &  particle-depleted | *Liquid N2 only* | 62 | m | IV | M1 | N0 | T3 | G2 | rectum | R2 | no | lung | 3.5 |
| 6 | CRC | whole, particle-concentrated &  particle-depleted | *Liquid N2 only* | 82 | m | IV | M1 | N2 | T3 | G2 | rectum | R2 | unknown | liver, colon transversum | 1.5 |
| 7 | CRC | whole, particle-concentrated &  particle-depleted | *RNA-later only* | 74 | f | IV | M1 | N2 | T3 | G2 | sigmoid colon | R2 | no | liver | 5 |
| 8 | CRC | whole, particle-concentrated &  particle-depleted | Liquid N2 & RNA-later | 88 | f | IV | M1 | N1 | T3 | G2 | sigmoid colon | R2 | no | liver, lung | 1 |
| 9 | CRC | whole, particle-concentrated &  particle-depleted | *Liquid N2 only* | 56 | m | IV | M1 | N2 | T4 | G2 | sigmoid colon | R2 | no | liver, peritoneal cavity | 3 |
| 10 | CRC | whole, particle-concentrated &  particle-depleted | Liquid N2 & RNA-later | 77 | f | IV | M1 | N1 | T4 | G2 | rectum | R2 | no | unknown | n.d. |

Radicality: residual tumor after surgery. CRC: colorectal cancer; n.d.: not determined

* Note: Biopsies of these patients were also tested in the validation stage

**Supplemental Table S2b: Patients’ (Serum and Tissue) and Controls’ (Serum) Samples Tested in the Validation Stage**

| **Sample no.** | **Type** | **Tested materials** | **Tissue Preservation** | **Age** | **Gender** | **UICC Stage** | **pM** | **pN** | **pT** | **Grade** | **Organ** | **Radicality** | **Neo-adjuvant treatment** | **Remarks** | **months survival post primary surgery** |
| --- | --- | --- | --- | --- | --- | --- | --- | --- | --- | --- | --- | --- | --- | --- | --- |
| 11 | CRC | Whole & particle-concentrated sera; tumor tissue | *Liquid N2 only* | 67 | m | IV | M1 | N1 | T3 | 2 | sigmoid  colon | R2 | 0 | carcinoma of the sigmoid colon + liver metastasis | 14 |
| 12 | CRC | Whole & particle-concentrated sera; tumor tissue | *Liquid N2 only* | 75 | f | II | M0 | N0 | T3 | 2 | colon | R0 | 0 | loc: 3 carcinomas in the colon descendens; carcinoma of the endometrium with peritoneal carcinosis | 6 |
| 13 | CRC | Whole & particle-concentrated sera; tumor tissue | *Liquid N2 only* | 62 | f | III | M0 | N2 | T3 | 3 | rectum | R0 | 0 | loc: rectum, distal third; 1994 uterus ca | 15 |
| 14 | CRC | Whole & particle-concentrated sera; tumor tissue | *Liquid N2 only* | 70 | m | III | M0 | N1 | T3 | 3 | rectum | R0 | 0 | loc: rectum, distal third; distant metastasis 2_2007: adrenal gland, lung, liver | 83 |
| 15 | CRC | Whole & particle-concentrated sera; tumor tissue | *Liquid N2 only* | 58 | m | III | M0 | N2 | T3 | 2 | rectum | R0 | 3 | also: chronic pancreatitis | 50 |
| 16 | CRC | Whole & particle-concentrated sera; tumor tissue | Liquid N2 & RNA-later | 69 | f | I | M0 | N0 | T2 | 2 | colon | R0 | 0 | Loc: colon ascendens | - |
| 17 | CRC | Whole & particle-concentrated sera; tumor tissue | *RNA-later only* | 42 | f | III | M0 | N2 | T2 | 3 | rectum | R0 | 2 | neuroendocrine tumor, L1 V0 | - |
| 18 | CRC | Whole & particle-concentrated sera; tumor tissue | *RNA-later only* | 89 | m | III | M0 | N1 | T3 | 2 | colon | R0 | 0 | - | 34 |
| 19 | CRC | Whole & particle-concentrated sera; tumor tissue | *Liquid N2 only* | 73 | f | II | M0 | N0 | T3 | 2 | Rectum | R0 | 0 | - | - |
| 20 | Healthy Control | Whole & particle-concentrated sera | - | 55 | m | - | - | - | - | - | - | - | - | - | - |
| 21 | Healthy Control | Whole & particle-concentrated sera | - | 41 | m | - | - | - | - | - | - | - | - | - | - |
| 22 | Healthy Control | Whole & particle-concentrated sera | - | 29 | f | - | - | - | - | - | - | - | - | - | - |
| 23 | Healthy Control | Whole & particle-concentrated sera | - | 35 | m | - | - | - | - | - | - | - | - | - | - |
| 24 | Healthy Control | Whole & particle-concentrated sera | - | 62 | m | - | - | - | - | - | - | - | - | - | - |
| 25 | Healthy Control | Whole & particle-concentrated sera | - | 34 | f | - | - | - | - | - | - | - | - | - | - |
| 26 | Healthy Control | Whole & particle-concentrated sera | - | 29 | m | - | - | - | - | - | - | - | - | - | - |
| 27 | Healthy Control | Whole & particle-concentrated sera | - | 35 | m | - | - | - | - | - | - | - | - | - | - |
| 28 | Healthy Control | Whole & particle-concentrated sera | - | 26 | m | - | - | - | - | - | - | - | - | - | - |
| 29 | Healthy Control | Whole & particle-concentrated sera | - | 32 | m | - | - | - | - | - | - | - | - | - | - |

CRC: colorectal cancer; Radicality: residual tumor after surgery.

As shown in Supplemental Table 2a and 2b, all the tested CRC patients were tested as whole sera (n=19), particle-concentrated sera (n=19), particle-depleted sera (n=10) and tumor tissue samples (n=25). The tumor tissues were preserved in frozen liquid nitrogen only (n=9), RNA-later only (n=4), and in both frozen liquid nitrogen and RNA-later (n=6). The latter overlapping tissue samples were considered for evaluating tissue preservation conditions for miRNA profiling (details in the main manuscript). Based on the results of the tissue samples, only the tissue sampled stored in liquid nitrogen (n=15) were considered for downstream analyses and comparisons. Ten healthy controls, tested as whole (n=10) and particle-concentrated sera (n=10), were also included in the analysis. In total, 93 RNA samples were investigated (see also Figure 1) in the current study (Tissue samples: 25; whole sera of CRC=19; whole sera of controls=10; particle-concentrated serum fractions of CRC= 19; particle-concentrated serum fractions of controls=10; particle-depleted serum fractions of CRC= 10).

Venous blood from CRC patients (in Supplemental Tables 1a and 1b) was collected from the central venous line under general anesthesia prior to operation. Blood donated by healthy individuals was aspirated from the medial cubital vein. All blood samples were collected in serum monovettes (Sarstedt, Nümbrecht, Germany), centrifuged at 2000 g, 10 min at room temperature and sera were subsequently stored at -80 ºC until use.

**Supplemental Table S2c: Patients’ and Control Individuals’ Serum Samples Tested in the Second Validation Stage**

| **Sample no.** | **Type** | **Tested materials** | **Serum-Pool** | **Age** | **Gender** | **UICC Stage** | **pM** | **pN** | **pT** | **Grade** | **Organ** |
| --- | --- | --- | --- | --- | --- | --- | --- | --- | --- | --- | --- |
| 30 | CRC | Particle-concentrated sera | 1 | 67 | m | II | M0 | N0 | T3 | 2 | Colon |
| 31 | CRC | Particle-concentrated sera | 1 | 77 | f | II | M0 | N0 | T3 | 2 | Sigmoid Colon |
| 32 | CRC | Particle-concentrated sera | 1 | 70 | m | II | M0 | N0 | T3 | 2 | Sigmoid Colon |
| 33 | CRC | Particle-concentrated sera | 1 | 62 | m | II | M0 | N0 | T3 | 3 | Sigmoid Colon |
| 34 | CRC | Particle-concentrated sera | 1 | 74 | f | II | M0 | N0 | T3 | 2 | Colon |
| 35 | CRC | Particle-concentrated sera | 1 | 74 | f | II | M0 | N0 | T3 | 3 | Colon |
| 36 | CRC | Particle-concentrated sera | 2 | 83 | m | II | M0 | N0 | T3 | 2 | Colon |
| 37 | CRC | Particle-concentrated sera | 2 | 86 | m | II | M0 | N0 | T3 | 2 | Sigmoid Colon |
| 38 | CRC | Particle-concentrated sera | 2 | 82 | m | II | M0 | N0 | T3 | 2 | Colon |
| 39 | CRC | Particle-concentrated sera | 2 | 82 | f | II | M0 | N0 | T3 | 3 | Colon |
| 40 | CRC | Particle-concentrated sera | 2 | 87 | f | II | M0 | N0 | T3 | 3 | Colon |
| 41 | CRC | Particle-concentrated sera | 2 | 82 | f | II | M0 | N0 | T4 | 3 | Colon |
| 42 | CRC | Particle-concentrated sera | 3 | 62 | m | II | M0 | N0 | T3 | 2 | Colon |
| 43 | CRC | Particle-concentrated sera | 3 | 59 | m | II | M0 | N0 | T3 | 2 | Colon |
| 44 | CRC | Particle-concentrated sera | 3 | 58 | m | II | M0 | N0 | T3 | 2 | Sigmoid Colon |
| 45 | CRC | Particle-concentrated sera | 3 | 45 | f | II | M0 | N0 | T4 | 3 | Colon |
| 46 | CRC | Particle-concentrated sera | 3 | 64 | f | II | M0 | N0 | T3 | 2 | Colon |
| 47 | CRC | Particle-concentrated sera | 3 | 68 | f | II | M0 | N0 | T3 | 2 | Colon |
| 48 | UC | Particle-concentrated sera | 4 | 61 | f | - | - | - | - | - | - |
| 49 | UC | Particle-concentrated sera | 4 | 61 | m | - | - | - | - | - | - |
| 50 | UC | Particle-concentrated sera | 4 | 62 | f | - | - | - | - | - | - |
| 51 | UC | Particle-concentrated sera | 4 | 63 | m | - | - | - | - | - | - |
| 52 | UC | Particle-concentrated sera | 4 | 65 | f | - | - | - | - | - | - |
| 53 | UC | Particle-concentrated sera | 4 | 66 | m | - | - | - | - | - | - |
| 54 | UC | Particle-concentrated sera | 5 | 17 | f | - | - | - | - | - | - |
| 55 | UC | Particle-concentrated sera | 5 | 22 | m | - | - | - | - | - | - |
| 56 | UC | Particle-concentrated sera | 5 | 21 | f | - | - | - | - | - | - |
| 57 | UC | Particle-concentrated sera | 5 | 20 | m | - | - | - | - | - | - |
| 58 | UC | Particle-concentrated sera | 5 | 18 | f | - | - | - | - | - | - |
| 59 | UC | Particle-concentrated sera | 5 | 20 | m | - | - | - | - | - | - |
| 60 | UC | Particle-concentrated sera | 6 | 43 | f | - | - | - | - | - | - |
| 61 | UC | Particle-concentrated sera | 6 | 45 | m | - | - | - | - | - | - |
| 62 | UC | Particle-concentrated sera | 6 | 48 | f | - | - | - | - | - | - |
| 63 | UC | Particle-concentrated sera | 6 | 44 | m | - | - | - | - | - | - |
| 64 | UC | Particle-concentrated sera | 6 | 48 | f | - | - | - | - | - | - |
| 65 | UC | Particle-concentrated sera | 6 | 42 | m | - | - | - | - | - | - |
| 66 | Healthy Control | Particle-concentrated sera | 7 | 45 | m | - | - | - | - | - | - |
| 67 | Healthy Control | Particle-concentrated sera | 7 | 23 | f | - | - | - | - | - | - |
| 68 | Healthy Control | Particle-concentrated sera | 7 | 30 | f | - | - | - | - | - | - |
| 69 | Healthy Control | Particle-concentrated sera | 7 | 37 | m | - | - | - | - | - | - |
| 70 | Healthy Control | Particle-concentrated sera | 7 | 64 | m | - | - | - | - | - | - |
| 71 | Healthy Control | Particle-concentrated sera | 7 | 57 | m | - | - | - | - | - | - |

CRC: colorectal cancer; UC: ulcerative colitis.

Venous blood from CRC patients (in Supplemental Tables 1c) was collected from the central venous line under general anesthesia prior to operation. Blood donated by healthy individuals and UC patients was aspirated from the medial cubital vein. All blood samples were collected in serum monovettes (Sarstedt, Nümbrecht, Germany), centrifuged at 2000 g, 10 min at room temperature and sera were subsequently stored at minimum at -80ºC until use.
