# Supplementary material for: Concentration of circulating miRNA-containing particles in serum enhances miRNA detection and reflects CRC tissue-related deregulations
Source: Oncotarget. 2016 Sep 23;7(46):75353–65. doi: 10.18632/oncotarget.12205 (PMC5342746; doi:10.18632/oncotarget.12205)
Supplement: Supplementary file 4 [file oncotarget-07-75353-s004.docx]

**Supplemental Table S7: The results of the ANOVA analysis across all the tested samples**

|  | | | **The calculated average of the normalized C_q_ values (dC_q_)** | | | | |  | | |
| --- | --- | --- | --- | --- | --- | --- | --- | --- | --- | --- |
| **miRNA Name** | **Count** | **Min Count** | **Concentrated-Sera-CRC** | **Concentrated-Sera-Control** | **Whole-Sera-Control** | **Whole-Sera-CRC** | **Tissue-**  **CRC** | **P-value** | **Rank** | **Benjamini-Hochberg corrected P- values** |
| hsa-miR-451a | 74 | 10 | 5.655310415 | 5.740874257 | 6.168972225 | 6.308920926 | -1.350568159 | 9.53×10^-41^ | 1 | 3.62×10^-39^ |
| hsa-miR-486-5p | 73 | 9 | 0.681208794 | 2.801025931 | 2.773375659 | 1.617603743 | -6.665843352 | 1.78×10^-33^ | 2 | 3.39×10^-32^ |
| hsa-miR-143-3p | 68 | 10 | -3.98322199 | -4.612713696 | -3.794511096 | -3.057788695 | 2.26764265 | 2.32×10^-23^ | 3 | 2.94×10^-22^ |
| hsa-miR-125b-5p | 58 | 8 | -4.22028341 | -5.464057197 | -4.659592808 | -3.256766984 | 1.24040848 | 1.18×10^-19^ | 4 | 1.12×10^-18^ |
| hsa-miR-23b-3p | 68 | 10 | -2.679760603 | -3.939285798 | -3.904634118 | -2.698865238 | 0.087046404 | 1.89×10^-19^ | 5 | 1.43×10^-18^ |
| hsa-miR-22-5p | 64 | 7 | 0.181775082 | -5.948867284 | -3.464393026 | 2.99042472 | -5.178653483 | 5.22×10^-15^ | 6 | 3.31×10^-14^ |
| hsa-miR-144-3p | 64 | 10 | -1.047667319 | -4.378616368 | -3.609283093 | -0.981075065 | -7.760712942 | 2.35×10^-14^ | 7 | 1.28×10^-13^ |
| hsa-miR-29c-3p | 58 | 9 | -2.949307666 | -5.432261806 | -5.093366066 | -3.07689916 | -1.210699545 | 5.66×10^-12^ | 8 | 2.69×10^-11^ |
| hsa-miR-23a-3p | 74 | 10 | 0.019339689 | -0.653767044 | -0.422776035 | -0.181577802 | 0.902224604 | 6.47×10^-12^ | 9 | 2.73×10^-11^ |
| hsa-miR-21-5p | 55 | 7 | -0.285525227 | -5.05157006 | -5.419246466 | -0.021301195 | 0.837739788 | 1.36×10^-11^ | 10 | 5.17×10^-11^ |
| hsa-miR-24-3p | 69 | 10 | -0.21734179 | -1.691093828 | -1.931740552 | -0.259046658 | 1.466670882 | 4.76×10^-11^ | 11 | 1.64×10^-10^ |
| hsa-miR-22-3p | 58 | 8 | -2.996906529 | -5.670335198 | -5.216357538 | -4.164866248 | -3.307971306 | 7.40×10^-11^ | 12 | 2.34×10^-10^ |
| hsa-miR-99a-5p | 63 | 10 | -3.778309357 | -4.539877585 | -4.58431907 | -4.059601131 | -1.291451334 | 3.96×10^-09^ | 13 | 1.15×10^-08^ |
| hsa-miR-423-5p | 61 | 7 | -2.50046231 | -5.017459123 | -5.843202664 | -1.832954039 | -3.553786665 | 5.17×10^-09^ | 14 | 1.40×10^-08^ |
| hsa-miR-15a-5p | 72 | 9 | 0.982308442 | 0.336878298 | -0.372596871 | 0.170862957 | -1.461719239 | 1.67×10^-08^ | 15 | 4.24×10^-08^ |
| hsa-miR-26a-5p | 52 | 7 | -1.555777141 | -5.891193907 | -5.133644432 | -2.335619493 | -1.654176612 | 4.78×10^-08^ | 16 | 1.14×10^-07^ |
| hsa-miR-93-5p | 73 | 10 | -0.1800431 | 0.771859105 | 0.558076652 | 0.433558567 | -0.669974322 | 5.25×10^-08^ | 17 | 1.17×10^-07^ |
| hsa-miR-365a-3p | 63 | 9 | -5.303578808 | -5.397612936 | -4.755551934 | -2.704168373 | -1.148481665 | 1.23×10^-07^ | 18 | 2.60×10^-07^ |
| hsa-miR-335-5p | 62 | 8 | -3.397107376 | -5.229992948 | -4.483350867 | -3.498286249 | -6.638419598 | 3.26×10^-07^ | 19 | 6.53×10^-07^ |
| hsa-miR-223-3p | 72 | 10 | 0.413384248 | -1.738610748 | -0.528107265 | 2.320070255 | -1.878818652 | 3.64×10^-07^ | 20 | 6.92×10^-07^ |
| hsa-miR-27b-3p | 59 | 8 | -2.281106578 | -2.98541656 | -2.940570254 | -2.590919009 | -0.897165871 | 1.77×10^-06^ | 21 | 3.22×10^-06^ |
| hsa-miR-101-3p | 64 | 9 | -1.935342212 | -5.27025064 | -4.058522109 | -1.951168151 | -3.280853584 | 9.69×10^-06^ | 22 | 1.67×10^-05^ |
| hsa-miR-320b | 62 | 9 | 1.01256527 | 0.216748424 | -0.177384088 | -0.283698646 | -0.090575597 | 0.000279065 | 23 | 0.000461064 |
| hsa-miR-342-3p | 73 | 10 | -2.93540921 | -1.729632192 | -0.865848289 | -1.913799568 | -1.408217979 | 0.000533282 | 24 | 0.000844363 |
| hsa-let-7f-5p | 47 | 4 | -4.249045583 | -5.577920261 | -6.33889378 | -4.980434435 | -3.183381339 | 0.000727389 | 25 | 0.001105632 |
| hsa-miR-15b-5p | 74 | 10 | -0.333520651 | -0.405864526 | -0.436059341 | -0.664554986 | -1.223084749 | 0.001603242 | 26 | 0.0023432 |
| hsa-miR-92a-3p | 74 | 10 | 0.950994097 | 2.077971289 | 1.651435421 | 1.957196761 | 0.974807559 | 0.002914699 | 27 | 0.004102169 |
| hsa-let-7d-5p | 58 | 8 | -4.636602996 | -5.146001632 | -5.520073716 | -4.526470024 | -3.532299443 | 0.003321743 | 28 | 0.004508079 |
| hsa-let-7i-5p | 70 | 10 | -3.568009803 | -2.793936483 | -3.486608824 | -3.038843925 | -2.087588296 | 0.006044861 | 29 | 0.007920852 |
| hsa-let-7d-3p | 49 | 6 | -4.110852073 | -2.104552156 | -2.293301072 | -3.560140516 | -2.560855915 | 0.008806521 | 30 | 0.011154927 |
| hsa-miR-126-3p | 69 | 10 | 1.044227204 | 0.977807293 | 0.61999467 | 0.4595585 | 0.100563158 | 0.01042866 | 31 | 0.012783519 |
| hsa-miR-146a-5p | 63 | 9 | -4.102545024 | -3.324564625 | -4.139173875 | -4.491594119 | -4.595317592 | 0.011086605 | 32 | 0.013165343 |
| hsa-miR-221-3p | 72 | 10 | -1.985179572 | -0.659193534 | -1.435933488 | -1.291689948 | -1.927787838 | 0.066533384 | 33 | 0.0766142 |
| hsa-miR-103a-3p | 73 | 10 | 0.186863423 | -0.133432671 | -0.180075882 | -0.261770816 | 0.274001012 | 0.096549266 | 34 | 0.107908003 |
| hsa-miR-107 | 74 | 10 | -0.636813135 | -1.018339718 | -0.792600045 | -1.111063973 | -0.653947415 | 0.212363548 | 35 | 0.230566138 |
| hsa-miR-424-5p | 51 | 6 | -1.287981094 | -1.523356679 | -1.283118578 | -1.885332969 | -2.003033952 | 0.296040625 | 36 | 0.312487326 |
| hsa-miR-151a-5p | 70 | 9 | -2.514571413 | -3.015633041 | -3.296739236 | -2.959700334 | -2.650915348 | 0.362014308 | 37 | 0.371798478 |
| hsa-miR-17-5p | 67 | 10 | -3.897502094 | -3.807568133 | -3.0432393 | -3.759938829 | -3.619853161 | 0.416729605 | 38 | 0.416729605 |
| hsa-miR-185-5p | 42 | 2 | -1.771778713 | -6.592039461 | -6.726977315 | -1.165736134 | -6.810503684 | 3.09181E-09 | - | - |
| hsa-miR-25-3p | 44 | 2 | -4.207156256 | -6.627373813 | -7.067116002 | -2.86075081 | -7.59731253 | 1.02605E-07 | - | - |
| hsa-miR-122-5p | 28 | 2 | -2.353735507 | -4.165794868 | -3.362311702 | -1.010699466 | -8.711721806 | 0.000299393 | - | - |
| hsa-miR-30b-5p | 45 | 2 | -2.551747019 | -4.344958799 | -6.070828865 | -2.413162243 | -4.793139785 | 0.002228456 | - | - |
| hsa-miR-1972 | 29 | 2 | -0.062682895 | -2.188161652 | -3.234607054 | -2.265525604 | -4.863212846 | 0.031494973 | - | - |
| hsa-miR-32-5p | 32 | 1 | -2.807190711 | -8.835649214 | -6.433060729 | -3.474579392 | -7.382954718 | 0.002873671 | - | - |
| hsa-miR-31-5p | 28 | 1 | -2.418998312 | -7.136456846 | -5.715810355 | -4.196145208 | -4.516970817 | 0.013596443 | - | - |
| hsa-miR-130a-3p | 36 | 0 | -3.321925622 | - | -3.884107268 | -4.243167021 | -6.071865011 | - | - | - |

In this table, the miRNAs have been sorted on basis of the P-values with the most significant expression on top.
