# Supplementary material for: Concentration of circulating miRNA-containing particles in serum enhances miRNA detection and reflects CRC tissue-related deregulations
Source: Oncotarget. 2016 Sep 23;7(46):75353–65. doi: 10.18632/oncotarget.12205 (PMC5342746; doi:10.18632/oncotarget.12205)
Supplement: Supplementary file 5 [file oncotarget-07-75353-s005.docx]

**Supplemental Table S8: Selected Results from the miEAA Analysis Tool of the Identified 22 miRNAs in the Particle-Concentrated CRC Sera**

| **Category** | **Subcategory** | **P-value FDR** | **P-value raw** | **miRNA** | **Enrichment** | **Expected** | **Observed** |
| --- | --- | --- | --- | --- | --- | --- | --- |
| **Immune cells Published** | **Exosomes** | 2.49×10^-10^ | 2.08×10^-10^ | hsa-miR-22-3p;hsa-miR-21-5p;hsa-miR-29c-3p;hsa-miR-101-3p;hsa-miR-23a-3p;hsa-miR-423-5p;hsa-miR-24-3p;hsa-let-7f-5p;hsa-miR-223-3p;hsa-miR-320b;hsa-miR-144-3p;hsa-miR-486-5p;hsa-miR-93-5p;hsa-miR-92a-3p;hsa-miR-146a-5p;hsa-miR-221-3p;hsa-let-7d-3p; | Over represented | 3.53109 | 17 |
| **Diseases miRWalk** | **Neoplasm_Metastasis** | 1.12×10^-05^ | 1.22×10^-07^ | hsa-miR-22-3p;hsa-miR-21-5p;hsa-miR-29c-3p;hsa-miR-101-3p;hsa-miR-23a-3p;hsa-miR-23b-3p;hsa-miR-24-3p;hsa-let-7f-5p;hsa-miR-125b-5p;hsa-miR-22-5p;hsa-miR-223-3p;hsa-miR-320b;hsa-miR-335-5p;hsa-miR-144-3p;hsa-miR-486-5p;hsa-miR-93-5p;hsa-miR-92a-3p;hsa-miR-146a-5p;hsa-miR-221-3p;hsa-let-7d-3p;hsa-miR-342-3p; | Over represented | 9.29096 | 21 |
| **Diseases miRWalk** | **Inflammation** | 0.0001 | 1.73×10^-05^ | hsa-miR-22-3p;hsa-miR-21-5p;hsa-miR-29c-3p;hsa-miR-101-3p;hsa-miR-24-3p;hsa-let-7f-5p;hsa-miR-125b-5p;hsa-miR-22-5p;hsa-miR-223-3p;hsa-miR-335-5p;hsa-miR-144-3p;hsa-miR-92a-3p;hsa-miR-146a-5p;hsa-miR-221-3p;hsa-let-7d-3p;hsa-miR-342-3p; | Over represented | 6.30791 | 16 |
| **Diseases miRWalk** | **Carcinoma** | 0.0002 | 2.71×10^-05^ | hsa-miR-22-3p;hsa-miR-21-5p;hsa-miR-29c-3p;hsa-miR-101-3p;hsa-miR-23a-3p;hsa-miR-23b-3p;hsa-miR-423-5p;hsa-miR-24-3p;hsa-let-7f-5p;hsa-miR-125b-5p;hsa-miR-22-5p;hsa-miR-223-3p;hsa-miR-320b;hsa-miR-335-5p;hsa-miR-144-3p;hsa-miR-486-5p;hsa-miR-93-5p;hsa-miR-92a-3p;hsa-miR-146a-5p;hsa-miR-221-3p;hsa-let-7d-3p;hsa-miR-342-3p; | Over represented | 13.7655 | 22 |
| **Diseases miRWalk** | **Adenocarcinoma** | 0.0002 | 2.81×10^-05^ | hsa-miR-22-3p;hsa-miR-21-5p;hsa-miR-101-3p;hsa-miR-23a-3p;hsa-miR-423-5p;hsa-miR-24-3p;hsa-let-7f-5p;hsa-miR-22-5p;hsa-miR-223-3p;hsa-miR-144-3p;hsa-miR-486-5p;hsa-miR-93-5p;hsa-miR-92a-3p;hsa-miR-146a-5p;hsa-miR-221-3p;hsa-let-7d-3p;hsa-miR-342-3p; | Over represented | 7.42655 | 17 |
| **Diseases miRWalk** | **Colonic_Neoplasms** | 0.0241 | 0.0117 | hsa-miR-22-3p;hsa-miR-21-5p;hsa-miR-423-5p;hsa-let-7f-5p;hsa-miR-22-5p;hsa-miR-335-5p;hsa-miR-93-5p;hsa-miR-92a-3p;hsa-miR-146a-5p;hsa-let-7d-3p; | Over represented | 4.87853 | 10 |
| **Diseases miRWalk** | **Colorectal_Neoplasms** | 0.1123 | 0.0944 | hsa-miR-21-5p;hsa-miR-23a-3p;hsa-miR-423-5p;hsa-miR-24-3p;hsa-let-7f-5p;hsa-miR-125b-5p;hsa-miR-223-3p;hsa-miR-335-5p;hsa-miR-486-5p;hsa-miR-92a-3p;hsa-miR-146a-5p;hsa-miR-221-3p;hsa-let-7d-3p;hsa-miR-342-3p; | Over represented | 10.4718 | 14 |
| **Organs miRWalk** | **Serum** | 1.60×10^-06^ | 3.50×10^-08^ | hsa-miR-22-3p;hsa-miR-21-5p;hsa-miR-29c-3p;hsa-miR-101-3p;hsa-miR-23a-3p;hsa-miR-23b-3p;hsa-miR-423-5p;hsa-miR-24-3p;hsa-let-7f-5p;hsa-miR-125b-5p;hsa-miR-22-5p;hsa-miR-223-3p;hsa-miR-335-5p;hsa-miR-144-3p;hsa-miR-486-5p;hsa-miR-93-5p;hsa-miR-92a-3p;hsa-miR-146a-5p;hsa-miR-221-3p;hsa-let-7d-3p; | Over represented | 7.55882 | 20 |
| **Organs miRWalk** | **Colon** | 0.0002 | 3.38×10^-05^ | hsa-miR-22-3p;hsa-miR-21-5p;hsa-miR-101-3p;hsa-miR-423-5p;hsa-let-7f-5p;hsa-miR-125b-5p;hsa-miR-22-5p;hsa-miR-223-3p;hsa-miR-335-5p;hsa-miR-486-5p;hsa-miR-93-5p;hsa-miR-92a-3p;hsa-miR-146a-5p;hsa-miR-221-3p;hsa-let-7d-3p; | Over represented | 5.76471 | 15 |
| **Pathways miRWalk** | **PTEN_PATHWAY_Biocarta** | 0.0001 | 8.05×10^-06^ | hsa-miR-22-3p;hsa-miR-21-5p;hsa-miR-29c-3p;hsa-miR-101-3p;hsa-miR-23a-3p;hsa-miR-23b-3p;hsa-miR-24-3p;hsa-let-7f-5p;hsa-miR-125b-5p;hsa-miR-22-5p;hsa-miR-223-3p;hsa-miR-335-5p;hsa-miR-144-3p;hsa-miR-486-5p;hsa-miR-93-5p;hsa-miR-92a-3p;hsa-miR-146a-5p;hsa-miR-221-3p;hsa-let-7d-3p;hsa-miR-342-3p; | Over represented | 10.0377 | 20 |
| **Pathways miRWalk** | **TCR_PATHWAY_Biocarta** | 0.0001 | 1.07×10^-05^ | hsa-miR-22-3p;hsa-miR-21-5p;hsa-miR-29c-3p;hsa-miR-101-3p;hsa-miR-23a-3p;hsa-miR-23b-3p;hsa-miR-423-5p;hsa-miR-24-3p;hsa-let-7f-5p;hsa-miR-125b-5p;hsa-miR-22-5p;hsa-miR-223-3p;hsa-miR-335-5p;hsa-miR-486-5p;hsa-miR-93-5p;hsa-miR-92a-3p;hsa-miR-146a-5p;hsa-miR-221-3p;hsa-let-7d-3p;hsa-miR-342-3p; | Over represented | 10.1887 | 20 |
| **Pathways miRWalk** | **APOPTOSIS_KEGG** | 0.0002 | 2.80×10^-05^ | hsa-miR-22-3p;hsa-miR-21-5p;hsa-miR-29c-3p;hsa-miR-101-3p;hsa-miR-23a-3p;hsa-miR-23b-3p;hsa-miR-423-5p;hsa-miR-24-3p;hsa-let-7f-5p;hsa-miR-125b-5p;hsa-miR-22-5p;hsa-miR-223-3p;hsa-miR-320b;hsa-miR-335-5p;hsa-miR-144-3p;hsa-miR-486-5p;hsa-miR-93-5p;hsa-miR-92a-3p;hsa-miR-146a-5p;hsa-miR-221-3p;hsa-let-7d-3p;hsa-miR-342-3p; | Over represented | 13.8113 | 22 |
| **Pathways miRWalk** | **MTOR_PATHWAY_Biocarta** | 0.0002 | 2.41×10^-05^ | hsa-miR-22-3p;hsa-miR-21-5p;hsa-miR-29c-3p;hsa-miR-101-3p;hsa-miR-23a-3p;hsa-miR-23b-3p;hsa-miR-24-3p;hsa-let-7f-5p;hsa-miR-125b-5p;hsa-miR-22-5p;hsa-miR-223-3p;hsa-miR-335-5p;hsa-miR-144-3p;hsa-miR-486-5p;hsa-miR-93-5p;hsa-miR-92a-3p;hsa-miR-146a-5p;hsa-miR-221-3p;hsa-let-7d-3p; | Over represented | 9.43396 | 19 |
| **Pathways miRWalk** | **RAS_PATHWAY_Biocarta** | 0.0002 | 2.78×10^-05^ | hsa-miR-22-3p;hsa-miR-21-5p;hsa-miR-29c-3p;hsa-miR-101-3p;hsa-miR-23a-3p;hsa-miR-23b-3p;hsa-miR-24-3p;hsa-let-7f-5p;hsa-miR-125b-5p;hsa-miR-22-5p;hsa-miR-223-3p;hsa-miR-335-5p;hsa-miR-486-5p;hsa-miR-93-5p;hsa-miR-92a-3p;hsa-miR-146a-5p;hsa-miR-221-3p;hsa-let-7d-3p;hsa-miR-342-3p; | Over represented | 9.50943 | 19 |
| **Pathways miRWalk** | **INFLAM_PATHWAY_Biocarta** | 0.0002 | 3.00×10^-05^ | hsa-miR-22-3p;hsa-miR-21-5p;hsa-miR-29c-3p;hsa-miR-101-3p;hsa-miR-23a-3p;hsa-miR-23b-3p;hsa-miR-423-5p;hsa-miR-24-3p;hsa-let-7f-5p;hsa-miR-125b-5p;hsa-miR-22-5p;hsa-miR-223-3p;hsa-miR-335-5p;hsa-miR-144-3p;hsa-miR-93-5p;hsa-miR-92a-3p;hsa-miR-146a-5p;hsa-miR-221-3p;hsa-let-7d-3p;hsa-miR-342-3p; | Over represented | 10.7547 | 20 |
| **Pathways miRWalk** | **RNA_PATHWAY_Biocarta** | 0.0002 | 4.49×10^-05^ | hsa-miR-22-3p;hsa-miR-21-5p;hsa-miR-29c-3p;hsa-miR-23a-3p;hsa-miR-23b-3p;hsa-miR-423-5p;hsa-miR-24-3p;hsa-let-7f-5p;hsa-miR-125b-5p;hsa-miR-22-5p;hsa-miR-223-3p;hsa-miR-320b;hsa-miR-335-5p;hsa-miR-93-5p;hsa-miR-92a-3p;hsa-miR-146a-5p;hsa-miR-221-3p;hsa-let-7d-3p;hsa-miR-342-3p; | Over represented | 9.77358 | 19 |
| **Pathways miRWalk** | **TOLL_LIKE_RECEPTOR_**  **SIGNALING_PATHWAY_KEGG** | 0.0005 | 0.0001 | hsa-miR-22-3p;hsa-miR-21-5p;hsa-miR-29c-3p;hsa-miR-101-3p;hsa-miR-23a-3p;hsa-miR-23b-3p;hsa-miR-423-5p;hsa-miR-24-3p;hsa-let-7f-5p;hsa-miR-125b-5p;hsa-miR-22-5p;hsa-miR-223-3p;hsa-miR-335-5p;hsa-miR-144-3p;hsa-miR-486-5p;hsa-miR-93-5p;hsa-miR-92a-3p;hsa-miR-146a-5p;hsa-miR-221-3p;hsa-let-7d-3p;hsa-miR-342-3p; | Over represented | 13.1698 | 21 |
| **Pathways miRWalk** | **IL6_PATHWAY_Biocarta** | 0.00065 | 0.0001 | hsa-miR-21-5p;hsa-miR-29c-3p;hsa-miR-101-3p;hsa-miR-23a-3p;hsa-miR-23b-3p;hsa-miR-423-5p;hsa-miR-24-3p;hsa-let-7f-5p;hsa-miR-125b-5p;hsa-miR-223-3p;hsa-miR-335-5p;hsa-miR-486-5p;hsa-miR-93-5p;hsa-miR-92a-3p;hsa-miR-146a-5p;hsa-miR-221-3p;hsa-let-7d-3p;hsa-miR-342-3p; | Over represented | 9.4717 | 18 |
| **Pathways miRWalk** | **STAT3_PATHWAY_Biocarta** | 0.0006 | 0.0001 | hsa-miR-22-3p;hsa-miR-21-5p;hsa-miR-101-3p;hsa-let-7f-5p;hsa-miR-125b-5p;hsa-miR-22-5p;hsa-miR-223-3p;hsa-miR-335-5p;hsa-miR-93-5p;hsa-miR-92a-3p;hsa-miR-146a-5p;hsa-miR-221-3p;hsa-let-7d-3p;hsa-miR-342-3p; | Over represented | 5.77358 | 14 |
| **Pathways miRWalk** | **CELL_CYCLE_KEGG** | 0.0009 | 0.0003 | hsa-miR-22-3p;hsa-miR-21-5p;hsa-miR-29c-3p;hsa-miR-101-3p;hsa-miR-23a-3p;hsa-miR-23b-3p;hsa-miR-423-5p;hsa-miR-24-3p;hsa-let-7f-5p;hsa-miR-125b-5p;hsa-miR-22-5p;hsa-miR-223-3p;hsa-miR-320b;hsa-miR-335-5p;hsa-miR-144-3p;hsa-miR-486-5p;hsa-miR-93-5p;hsa-miR-92a-3p;hsa-miR-146a-5p;hsa-miR-221-3p;hsa-let-7d-3p;hsa-miR-342-3p; | Over represented | 15.4717 | 22 |
| **Pathways miRWalk** | **P53_PATHWAY_Biocarta** | 0.0009 | 0.0003 | hsa-miR-22-3p;hsa-miR-21-5p;hsa-miR-29c-3p;hsa-miR-101-3p;hsa-miR-23a-3p;hsa-miR-23b-3p;hsa-miR-423-5p;hsa-miR-24-3p;hsa-let-7f-5p;hsa-miR-125b-5p;hsa-miR-22-5p;hsa-miR-223-3p;hsa-miR-320b;hsa-miR-335-5p;hsa-miR-144-3p;hsa-miR-93-5p;hsa-miR-92a-3p;hsa-miR-146a-5p;hsa-miR-221-3p;hsa-let-7d-3p;hsa-miR-342-3p; | Over represented | 13.7358 | 21 |
| **Pathways miRWalk** | **NFKB_PATHWAY_Biocarta** | 0.0012 | 0.0005 | hsa-miR-22-3p;hsa-miR-21-5p;hsa-miR-29c-3p;hsa-miR-23a-3p;hsa-miR-23b-3p;hsa-miR-24-3p;hsa-let-7f-5p;hsa-miR-125b-5p;hsa-miR-22-5p;hsa-miR-223-3p;hsa-miR-144-3p;hsa-miR-92a-3p;hsa-miR-146a-5p;hsa-miR-221-3p;hsa-let-7d-3p;hsa-miR-342-3p; | Over represented | 8.11321 | 16 |
| **Pathways miRWalk** | **CYTOKINE_CYTOKINE_**  **RECEPTOR_**  **INTERACTION_KEGG** | 0.0014 | 0.0006 | hsa-miR-22-3p;hsa-miR-21-5p;hsa-miR-29c-3p;hsa-miR-101-3p;hsa-miR-23a-3p;hsa-miR-23b-3p;hsa-miR-423-5p;hsa-miR-24-3p;hsa-let-7f-5p;hsa-miR-125b-5p;hsa-miR-22-5p;hsa-miR-223-3p;hsa-miR-335-5p;hsa-miR-144-3p;hsa-miR-486-5p;hsa-miR-93-5p;hsa-miR-92a-3p;hsa-miR-146a-5p;hsa-miR-221-3p;hsa-let-7d-3p;hsa-miR-342-3p; | Over represented | 14.0755 | 21 |
| **Pathways miRWalk** | **COLORECTAL_CANCER_KEGG** | 0.0019 | 0.0009 | hsa-miR-22-3p;hsa-miR-21-5p;hsa-miR-29c-3p;hsa-miR-101-3p;hsa-miR-23a-3p;hsa-miR-23b-3p;hsa-miR-423-5p;hsa-miR-24-3p;hsa-let-7f-5p;hsa-miR-125b-5p;hsa-miR-22-5p;hsa-miR-223-3p;hsa-miR-320b;hsa-miR-335-5p;hsa-miR-144-3p;hsa-miR-486-5p;hsa-miR-93-5p;hsa-miR-92a-3p;hsa-miR-146a-5p;hsa-miR-221-3p;hsa-let-7d-3p;hsa-miR-342-3p; | Over represented | 16.1132 | 22 |
| **Pathways miRWalk** | **WNT_SIGNALING_**  **PATHWAY_KEGG** | 0.0026 | 0.0013 | hsa-miR-22-3p;hsa-miR-21-5p;hsa-miR-29c-3p;hsa-miR-101-3p;hsa-miR-23a-3p;hsa-miR-23b-3p;hsa-miR-423-5p;hsa-miR-24-3p;hsa-let-7f-5p;hsa-miR-125b-5p;hsa-miR-22-5p;hsa-miR-223-3p;hsa-miR-320b;hsa-miR-335-5p;hsa-miR-144-3p;hsa-miR-486-5p;hsa-miR-93-5p;hsa-miR-92a-3p;hsa-miR-146a-5p;hsa-miR-221-3p;hsa-let-7d-3p; | Over represented | 14.6415 | 21 |
| **Pathways miRWalk** | **ANTIGEN_PROCESSING_AND_**  **PRESENTATION_KEGG** | 0.0044 | 0.0025 | hsa-miR-22-3p;hsa-miR-21-5p;hsa-miR-29c-3p;hsa-miR-101-3p;hsa-miR-23a-3p;hsa-miR-23b-3p;hsa-miR-24-3p;hsa-let-7f-5p;hsa-miR-125b-5p;hsa-miR-22-5p;hsa-miR-223-3p;hsa-miR-93-5p;hsa-miR-92a-3p;hsa-miR-146a-5p;hsa-miR-221-3p;hsa-let-7d-3p;hsa-miR-342-3p; | Over represented | 10.1509 | 17 |
| **Pathways miRWalk** | **JAK_STAT_SIGNALING_**  **PATHWAY_KEGG** | 0.0062 | 0.0037 | hsa-miR-22-3p;hsa-miR-21-5p;hsa-miR-29c-3p;hsa-miR-101-3p;hsa-miR-23a-3p;hsa-miR-23b-3p;hsa-miR-24-3p;hsa-let-7f-5p;hsa-miR-125b-5p;hsa-miR-22-5p;hsa-miR-223-3p;hsa-miR-335-5p;hsa-miR-144-3p;hsa-miR-486-5p;hsa-miR-93-5p;hsa-miR-92a-3p;hsa-miR-146a-5p;hsa-miR-221-3p;hsa-let-7d-3p;hsa-miR-342-3p; | Over represented | 14 | 20 |

The miRWalk “validated target” database is constructed from a basic word-search algorithm that sometimes returns co-occurrence results without biological implications. We have thus manually inspected all available studies (in PubMed) to confirm a potential functional interplay of our miRNA candidates with key inflammatory-/cancer-related factors, such as IL6, STAT3 and NF-κB, as shown in Supplemental Tables 9 and 11.
