# Supplementary material for: Concentration of circulating miRNA-containing particles in serum enhances miRNA detection and reflects CRC tissue-related deregulations
Source: Oncotarget. 2016 Sep 23;7(46):75353–65. doi: 10.18632/oncotarget.12205 (PMC5342746; doi:10.18632/oncotarget.12205)
Supplement: Supplementary file 6 [file oncotarget-07-75353-s006.docx]

**Supplemental Table S9: Possible Interplay of the Identified 22 miRNAs in the Particle-Concentrated Sera with Key Inflammatory and Cancer-Related Pathways. A complete list of citations shown here can be found in the References section on pp. 4-6 of the supplementary pdf file.**

|  | | **I. Particle-Concentrated Sera (CRC)**  ***vs.* Particle-Concentrated Sera (Controls)** ^a^ | |  | **Consistent Expression Patterns of I & II^§^ (15/22; 68%)** ^b^ | **Consistent Expression Patterns of I & III ^§^ (20/22; 91%)** ^c^ |  | **IL6/STAT/ NF-κB (15/22; 68%)** | Selected Studies/Citations |  |
| --- | --- | --- | --- | --- | --- | --- | --- | --- | --- | --- |
| Expression Pattern with respect to CRC | miRNA Candidates | Fold Change | Adjusted P-value |  |  |  |  |  |  |  |
| **↑ Particle-Concentrated Sera & Tissue** (n=10) | hsa-miR-22-3p | 2.673 | 3.04×10^-05^ |  | **Yes** | **Yes** |  | - | - |  |
|  | hsa-miR-21-5p | 4.766 | 5.65×10^-05^ |  | **Yes** | **Yes** |  | **Yes** | [17]; [18]; [19] |  |
|  | hsa-miR-29c-3p | 2.482 | 5.65×10^-05^ |  | **Yes** | **Yes** |  | **Yes** | [18]; [20] |  |
|  | hsa-miR-101-3p | 3.334 | 8.91×10^-05^ |  | **Yes** | **Yes** |  | **Yes** | [21] |  |
|  | hsa-miR-23a-3p | 0.673 | 0.0002 |  | **Yes** | **Yes** |  | **Yes** | [22] |  |
|  | hsa-miR-23b-3p | 1.259 | 0.0004 |  | **Yes** | **Yes** |  | **Yes** | [23]; [24] |  |
|  | hsa-miR-423-5p | 2.516 | 0.0010 |  | **Yes** | **Yes** |  | - | - |  |
|  | hsa-miR-24-3p | 1.473 | 0.00293 |  | **Yes** | **Yes** |  | **Yes** | [24]; [25] |  |
|  | hsa-let-7f-5p | 1.328 | 0.0095 |  | **Yes** | **Yes** |  | **Yes** | [26]; [27]; [21] |  |
|  | hsa-miR-125b-5p | 1.243 | 0.0454 |  | **Yes** | **Yes** |  | **Yes** | [28]; [24]; [29]; [30] |  |
|  | | | | | | | | | | |
| **↑ Particle-Concentrated Sera Only** (n=3) | hsa-miR-22-5p | 6.130 | 7.40×10^-05^ |  | No | **Yes** |  | - | - |  |
|  | hsa-miR-223-3p | 2.151 | 0.0051 |  | No | **Yes** |  | **Yes** | [31]; [32] |  |
|  | hsa-miR-320b | 0.795 | 0.0134 |  | No | **Yes** |  | - | - |  |
|  | | | | | | | | | | |
| **↑ Particle-Concentrated Sera & ↓Tissue** (n=2) | hsa-miR-335-5p | 1.832 | 0.0002 |  | No | No |  | **Yes** | [33] |  |
|  | hsa-miR-144-3p | 3.330 | 0.0004 |  | No | No |  | - | - |  |
|  | | | | | | | | | | |
| **↓ Particle-Concentrated Sera & Tissue** (n=5) | hsa-miR-486-5p | -2.119 | 0.0001 |  | **Yes** | **Yes** |  | - | - |  |
|  | hsa-miR-93-5p | -0.951 | 0.0003 |  | **Yes** | **Yes** |  | **Yes** | [34]; [35] |  |
|  | hsa-miR-92a-3p | -1.1269 | 0.0026 |  | **Yes** | **Yes** |  | **Yes** | [24] |  |
|  | hsa-miR-146a-5p | -0.777 | 0.0030 |  | **Yes** | **Yes** |  | **Yes** | [18];[36]; [37]; [38] |  |
|  | hsa-miR-221-3p | -1.325 | 0.0064 |  | **Yes** | **Yes** |  | **Yes** | [39]; [40]; [41] |  |
|  | | | | | | | | | | |
| **↓ Particle-Concentrated Sera only** (n=2) | hsa-let-7d-3p | -2.006 | 0.00070 |  | No | Yes |  | **Yes** | [26]; [27]; [21] |  |
|  | hsa-miR-342-3p | -1.205 | 0.0064 |  | No | Yes |  | - | - |  |

hsa:homo sapiens; ↑: upregulated; ↓: downregulated; ^a^: Fold changes (ddCq values of particle-concentrated CRC sera minus those of the controls); positive values: upregulation in particle-concentrated CRC sera; ^b,c, §^: Details can be found in Table 1 in the main manuscript and in Supplemental Data.
