# Supplementary material for: Concentration of circulating miRNA-containing particles in serum enhances miRNA detection and reflects CRC tissue-related deregulations
Source: Oncotarget. 2016 Sep 23;7(46):75353–65. doi: 10.18632/oncotarget.12205 (PMC5342746; doi:10.18632/oncotarget.12205)
Supplement: Supplementary file 7 [file oncotarget-07-75353-s007.docx]

**Supplemental Table S10: Particle-Concentrated Serum miRNA Patterns of CRC Patients with Metastasis Reflect Tissue-Related Expression**

|  |  | **IV. Particle-Concentrated Sera (M1-CRC)**  ***vs.* Particle-Concentrated Sera (Controls)** ^a^ | |  | **V. Particle-Concentrated Sera (M1-CRC) *vs.* Tissue-M1-CRC**^b^ | | **Consistent Expression Patterns of 4 & 5 (15/27:56% )** |  | | **VI. Tissue-M1-CRC**  ***vs.* Particle-Concentrated Sera (Controls)** ^c^ | | | | **Consistent Expression Patterns of 4 & 6**  **(27/27: 100%)** | |  |
| --- | --- | --- | --- | --- | --- | --- | --- | --- | --- | --- | --- | --- | --- | --- | --- | --- |
| Expression Pattern with respect to CRC | miRNA Candidates | Fold Change^a^ | Adjusted P-value |  | Fold Change^b^ | Adjusted P-value |  |  |  | Fold Change^c^ | | Adjusted P-value | |  |  |  |
| **↑ Particle-Concentrated Sera & Tissue (10 +2)** | hsa-miR-22-3p | 2.964 | 1.40×10^-06^ |  | 0.215 | *0.7759* | **Yes** |  |  | 2.749 | | 0.0001 | | **Yes** | |  |
|  | hsa-miR-21-5p | 5.109 | 3.31×10^-06^ |  | -1.71 | *0.2459* | **Yes** |  |  | 6.827 | | 0.0005 | | **Yes** | |  |
|  | hsa-miR-29c-3p | 2.823 | 1.98×10^-06^ |  | -2.115 | 0.0340 | **Yes** |  |  | 4.939 | | 0.0004 | | **Yes** | |  |
|  | hsa-miR-101-3p | 3.877 | 3.31×10^-06^ |  | 0.773 | *0.5755* | **Yes** |  |  | 3.103 | | 0.0289 | | **Yes** | |  |
|  | hsa-miR-23a-3p | 0.782 | 0.0003 |  | -0.896 | 0.0384 | **Yes** |  |  | 1.678 | | 0.0020 | | **Yes** | |  |
|  | hsa-miR-23b-3p | 1.794 | 3.22×10^-06^ |  | -2.621 | 3.62×10^-06^ | **Yes** |  |  | 4.415 | | 2.40×10^-08^ | | **Yes** | |  |
|  | hsa-miR-423-5p | 3.748 | 1.44×10^-07^ |  | 2.214 | 0.0011 | No |  |  | 1.533 | | 0.0119 | | **Yes** | |  |
|  | hsa-miR-24-3p | 2.411 | 1.44×10^-07^ |  | -1.101 | 0.0398 | **Yes** |  |  | 3.512 | | 4.90×10^-05^ | | **Yes** | |  |
|  | hsa-let-7f-5p | 1.253 | 0.0160 |  | -1.882 | *0.1237* | **Yes** |  |  | 3.136 | | 0.0232 | | **Yes** | |  |
|  | hsa-miR-125b-5p | 0.672 | 0.0737 |  | -6.046 | 1.23×10^-06^ | **Yes** |  |  | 6.719 | | 2.51×10^-08^ | | **Yes** | |  |
|  | **hsa-miR-26a-5p** | 4.587 | 0.0004 |  | -0.414 | *0.7926* | **Yes** |  |  | 5.002 | | 0.0034 | | **Yes** | |  |
|  | **hsa-miR-25-3p** | 2.420 | 0.0006 |  | 2.393 | *0.1151* | **Yes** |  |  | 0.026 | | *0.9830* | | **Yes** | |  |
|  | | | | | | | | | | | | | | |  |  |
| **↑ Particle-Concentrated Sera Only (3+2)** | hsa-miR-22-5p | 7.717 | 4.28×10^-07^ |  | 6.485 | 3.62×10^-06^ | No | |  | | 1.231 | | 0.0865 | **Yes** | | |
|  | hsa-miR-223-3p | 3.940 | 8.42×10^-08^ |  | 3.626 | 0.0082 | No | |  |  | 0.314 | | *0.7933* | **Yes** | | |
|  | hsa-miR-320b | 0.754 | 0.0561 |  | 1.225 | 0.0520 | No | |  |  | -0.470 | | *0.4111* | **Yes** | | |
|  | **hsa-miR-15a-5p** | 1.057 | 0.0042 |  | 2.250 | 0.0113 | No | |  |  | -1.192 | | *0.1322* | **Yes** | | |
|  | **hsa-miR-185-5p** | 4.820 | 0.0104 |  | 4.291 | 0.0042 | No | |  |  | 0.528 | | *0.7378* | **Yes** | | |
|  | | | | | | | | | | | | | | |  |  |
| **↑ Particle-Concentrated Sera & ↓Tissue (2)** | hsa-miR-335-5p | 1.690 | 0.0004 |  | 2.679 | 0.0014 | No | |  | | -0.989 | | *0.1322* | **Yes** | | |
|  | hsa-miR-144-3p | 4.455 | 5.27×10^-06^ |  | 6.115 | 0.0025 | No | |  |  | -1.659 | | *0.2234* | **Yes** | | |
|  | | | | | | | | | | | | | | |  |  |
| **↓ Particle-Concentrated Sera & Tissue (5)** | hsa-miR-486-5p | -3.0154 | 1.81×10^-05^ |  | 7.091 | 1.47×10^-08^ | **Yes** | |  | | -10.106 | | 3.98×10^-09^ | **Yes** | | |
|  | hsa-miR-93-5p | -1.422 | 3.22×10^-06^ |  | -0.099 | *0.7926* | **Yes** | |  |  | -1.322 | | 0.0005 | **Yes** | | |
|  | hsa-miR-92a-3p | -0.867 | 0.0174 |  | 0.050 | *0.9596* | **Yes** | |  |  | -0.918 | | *0.1137* | **Yes** | | |
|  | hsa-miR-146a-5p | -1.257 | 1.34×10^-05^ |  | 0.0209 | *0.9596* | **Yes** | |  |  | -1.278 | | 0.0232 | **Yes** | | |
|  | hsa-miR-221-3p | -2.401 | 3.31×10^-06^ |  | -1.769 | 0.0041 | No | |  |  | -0.631 | | *0.1769* | **Yes** | | |
|  | | | | | | | | | | | | | | |  |  |
| **↓ Particle-Concentrated Sera Only (2+1)** | hsa-let-7d-3p | -2.709 | 5.27×10^-06^ |  | -2.262 | 0.0002 | No | |  | | -0.447 | | *0.2771* | **Yes** | | |
|  | hsa-miR-342-3p | -1.752 | 0.0019 |  | -1.885 | 0.0042 | No | |  |  | 0.13345671 | | *0.7955* | **Yes** | | |
|  | **hsa-let-7i-5p** | -1.610 | 0.0005 |  | -2.898 | 0.0042 | No | |  |  | 1.288579676 | | *0.1200* | **Yes** | | |

hsa: *homo* *sapiens*; ↑: upregulated; ↓: downregulated; M1: CRC with metastasis; **Yes**: consistent deregulation; Adjusted P-value: t-test p-value after adjustment for multiple testing

**miRNAs in bold prints**: Six miRNAs were additionally detected, to be differentially deregulated, when comparing samples with metastasis (M1). The other miRNAs (n=22) are the same shown in Table 1 in the main manuscript. Because of a technical detection problem of miR-1972, the results of this miRNA are not shown in this Table. Thus, a total of 27 miRNAs instead of 28 miRNAs are shown here. miR-125b and miR-320 showed border significance in particle-concentrated sera of metastatic CRC while being highly significant in tissue CRC samples.

^a^: These fold changes represent the ddC_q_ values of particle-concentrated sera of metastatic CRC minus those of the controls. While positives ddC_q_ values here indicate up-regulation in particle-concentrated sera of metastatic CRC, negative values indicate down-regulations in the particle-concentrated sera of metastatic CRC compared to the corresponding control serum samples.

^b^: These fold changes represent the ddC_q_ values of particle-concentrated sera of metastatic CRC minus those of the metastatic CRC tissues. While positives ddC_q_ values here indicate up-regulation in the particle-concentrated sera of metastatic CRC (or downregulations in the metastatic CRC tissues), negative values indicate down-regulations in particle-concentrated sera of metastatic CRC (or up-regulations in the metastatic CRC tissues).

^c^: These fold changes represent the ddC**q** values of particle-concentrated sera of metastatic CRC minus those of the corresponding control sera. While positives ddCq values here indicate up-regulation in the metastatic CRC tissues, negative values indicate down-regulations in metastatic CRC tissues compared to particle-concentrated sera of controls.
