# Supplementary material for: Concentration of circulating miRNA-containing particles in serum enhances miRNA detection and reflects CRC tissue-related deregulations
Source: Oncotarget. 2016 Sep 23;7(46):75353–65. doi: 10.18632/oncotarget.12205 (PMC5342746; doi:10.18632/oncotarget.12205)
Supplement: Supplementary file 8 [file oncotarget-07-75353-s008.docx]

**Supplemental Table S11: Possible Interplay of the Identified 22 miRNAs in the Particle-Concentrated Sera of Metastatic CRC Patients with Key Inflammatory and Cancer-Related Pathways. A complete list of citations shown here can be found in the References section on pp. 4-6 of the supplementary pdf file.**

|  |  | **IV. Particle-Concentrated Sera (M1-CRC) *vs.* Particle-Concentrated Sera (Controls)** ^a^ | |  | **Consistent Expression Patterns of IV & V (15/27:56% )** ^b^ | **Consistent Expression Patterns of IV & VI (27/27: 100%)** ^c^ |  | **IL6/STAT/ NF-κB (16/27; 59%)** | | **Selected Studies/ Citations** |  |
| --- | --- | --- | --- | --- | --- | --- | --- | --- | --- | --- | --- |
| Expression Pattern with respect to CRC | miRNA Candidates | Fold Change | Adjusted P-value |  |  |  |  |  |  |  |  |
| **↑ Particle-Concentrated Sera & Tissue (10 +2)** | hsa-miR-22-3p | 2.964 | 1.40×10^-06^ |  | **Yes** | **Yes** |  | - | | - |  |
|  | hsa-miR-21-5p | 5.109 | 3.31×10^-06^ |  | **Yes** | **Yes** |  | **Yes** | | [17]; [18]; [19] |  |
|  | hsa-miR-29c-3p | 2.823 | 1.98×10^-06^ |  | **Yes** | **Yes** |  | **Yes** | | [18]; [20] |  |
|  | hsa-miR-101-3p | 3.877 | 3.31×10^-06^ |  | **Yes** | **Yes** |  | **Yes** | | [21] |  |
|  | hsa-miR-23a-3p | 0.782 | 0.0003 |  | **Yes** | **Yes** |  | **Yes** | | [22] |  |
|  | hsa-miR-23b-3p | 1.794 | 3.22×10^-06^ |  | **Yes** | **Yes** |  | **Yes** | | [23]; [24] |  |
|  | hsa-miR-423-5p | 3.748 | 1.44×10^-07^ |  | No | **Yes** |  | - | | - |  |
|  | hsa-miR-24-3p | 2.411 | 1.44×10^-07^ |  | **Yes** | **Yes** |  | **Yes** | | [24]; [25] |  |
|  | hsa-let-7f-5p | 1.253 | 0.0160 |  | **Yes** | **Yes** |  | **Yes** | | [26]; [27]; [21] |  |
|  | hsa-miR-125b-5p | 0.672 | 0.0737 |  | **Yes** | **Yes** |  | **Yes** | | [28]; [24]; [29]; [30] |  |
|  | **hsa-miR-26a-5p** | 4.587 | 0.0004 |  | **Yes** | **Yes** |  | **Yes** | | [42, 43] |  |
|  | **hsa-miR-25-3p** | 2.420 | 0.0006 |  | **Yes** | **Yes** |  | - | | - |  |
|  | | | | | | | | | | | |
| **↑ Particle-Concentrated Sera Only (3+2)** | hsa-miR-22-5p | 7.717 | 4.28×10^-07^ |  | No | **Yes** |  | - | - | |  |
|  | hsa-miR-223-3p | 3.940 | 8.42×10^-08^ |  | No | **Yes** |  | **Yes** | [31]; [32] | |  |
|  | hsa-miR-320b | 0.754 | 0.0561 |  | No | **Yes** |  | - | - | |  |
|  | **hsa-miR-15a-5p** | 1.057 | 0.0042 |  | No | **Yes** |  | - | *-* | |  |
|  | **hsa-miR-185-5p** | 4.820 | 0.0104 |  | No | **Yes** |  | - | *-* | |  |
|  | | | | | | | | | | | |
| **↑ Particle-Concentrated Sera & ↓Tissue (2)** | hsa-miR-335-5p | 1.690 | 0.0004 |  | No | **Yes** |  | **Yes** | [33] | |  |
|  | hsa-miR-144-3p | 4.455 | 5.27×10^-06^ |  | No | **Yes** |  | - | - | |  |
|  | | | | | | | | | | | |
| **↓ Particle-Concentrated Sera & Tissue (5)** | hsa-miR-486-5p | -3.0154 | 1.81×10^-05^ |  | **Yes** | **Yes** |  | - | - | |  |
|  | hsa-miR-93-5p | -1.422 | 3.22×10^-06^ |  | **Yes** | **Yes** |  | **Yes** | [34]; [35] | |  |
|  | hsa-miR-92a-3p | -0.867 | 0.0174 |  | **Yes** | **Yes** |  | **Yes** | [24] | |  |
|  | hsa-miR-146a-5p | -1.257 | 1.34×10^-05^ |  | **Yes** | **Yes** |  | **Yes** | [18];[36]; [37]; [38] | |  |
|  | hsa-miR-221-3p | -2.401 | 3.31×10^-06^ |  | No | **Yes** |  | **Yes** | [39]; [40]; [41] | |  |
|  | | | | | | | | | | | |
| **↓ Particle-Concentrated Sera only (2+1)** | hsa-let-7d-3p | -2.709 | 5.27×10^-06^ |  | No | **Yes** |  | **Yes** | [26]; [27]; [21] | |  |
|  | hsa-miR-342-3p | -1.752 | 0.0019 |  | No | **Yes** |  | - | - | |  |
|  | **hsa-let-7i-5p** | -1.610 | 0.0005 |  | No | **Yes** |  | - | - | |  |

hsa: *homo* *sapiens*; ↑: upregulated; ↓: downregulated; M1: CRC with metastasis; Adjusted P-value: t-test P-value after adjustment for multiple testing.

**miRNAs in bold prints**: Six miRNAs were additionally detected, to be differentially deregulated, when comparing samples with metastasis (M1). The other miRNAs (n=22) are the same shown in Table 1 in the main manuscript. Because of a technical detection problem of miR-1972, the results of this miRNA are not shown in this Table. Thus, a total of 27 miRNAs instead of 28 miRNAs are shown here. miR-125b and miR-320 showed border significance in particle-concentrated of sera of metastatic CRC (M1-CRC) while being highly significant in tissue CRC samples.

^a^: These fold changes represent the ddC_q_ values of particle-concentrated of sera of metastatic CRC (M1-CRC) minus those of the particle-concentrated of sera of controls. While positives ddC_q_ values here indicate up-regulation in particle-concentrated of sera of metastatic CRC (M1-CRC), negative values indicate down-regulations in the particle-concentrated of sera of metastatic CRC (M1-CRC) compared to the particle-concentrated of sera of control samples.

^b,c^: Details can be found in Supplemental Table 10.
